# Heavy Atom as a Molecular Sensor of Electronic Density: The Advanced Dimer-Type Light-Emitting System for NIR Emission

**DOI:** 10.1021/acsami.4c21674

**Published:** 2025-01-31

**Authors:** Michał Mońka, Piotr Pander, Daria Grzywacz, Artur Sikorski, Radosław Rogowski, Piotr Bojarski, Andrew P. Monkman, Illia E. Serdiuk

**Affiliations:** † Faculty of Mathematics, Physics and Informatics, University of Gdańsk, Wita Stwosza 57, 80-308 Gdańsk, Poland; ‡ Faculty of Chemistry, Silesian University of Technology, M. Strzody 9, 44-100 Gliwice, Poland; § Centre for Organic and Nanohybrid Electronics, Silesian University of Technology, Konarskiego 22B, 44-100 Gliwice, Poland; ∥ Faculty of Chemistry, University of Gdansk, Wita Stwosza 63, 80-308 Gdańsk, Poland; ⊥ Physics Department, Durham University, South Road, Durham DH1 3LE, U.K.

**Keywords:** heavy-atom effect, TADF, OLED, NIR emission, aggregation, π–π stacking

## Abstract

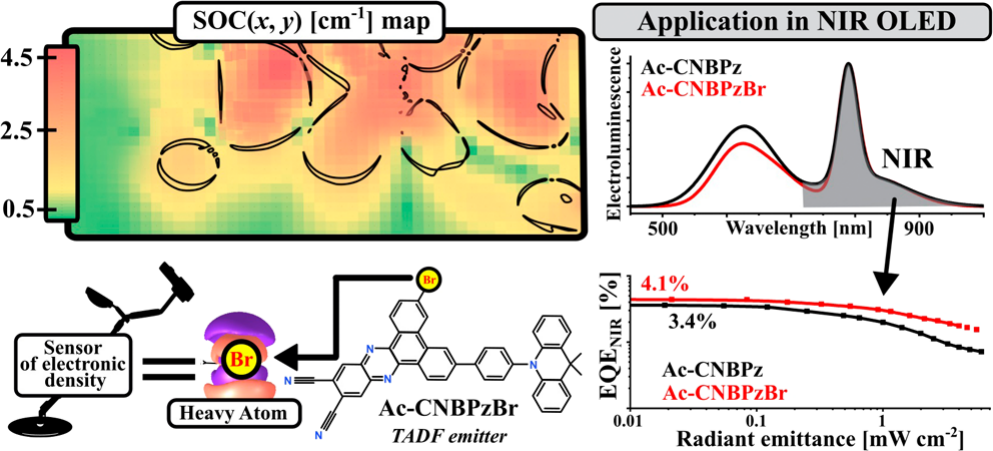

The approaches to design and control intermolecular interactions for a selective enhancement of specific process(es) are of high interest in technologies using molecular materials. Here, we describe how π–π stacking enables control over the heavy-atom effect and spin–orbit coupling (SOC) through dimerization of an organic emitter in solid media. π–π interactions in a red thermally activated delayed fluorescence (TADF) emitter **Ac-CNBPz** afford specific types of dimers. In its brominated derivative **Ac-CNBPzBr**, the vicinity of the Br atom and the electronic density of the dimer involved in a spin-flip transition afford up to 200-fold increase of the SOC, in the most favorable case, attributed to the external heavy-atom effect (EHAE) of the halogen atom. The presence of such dimers in the films of **Ac-CNBPzBr** provides enhancement of reverse intersystem crossing, and thus, TADF occurs mostly within a few microseconds, up to 20 times faster than in **Ac-CNBPz**. For this reason, organic light-emitting diodes using **Ac-CNBPzBr** as an emitter and an assistant dopant show a decreased efficiency roll-off by a factor of 4 and 1.5, respectively. The crucial aspects of the intermolecular electronic interactions between a chromophore system and an HA together with the particularly favorable dimer geometry not only help to understand the nature of the EHAE but also provide guidelines for the molecular design of emitters for all-organic light-emitting devices with enhanced stability.

## Introduction

Intermolecular interactions can strongly alter the properties of materials in condensed phase. π–π interactions and stacking^1^ are particularly significant for the planar (poly)aromatic molecules applied in optoelectronics as they have strong effect on the π–π* electronic transitions. Such interactions are one of the driving forces of the formation of aggregated species, including dimers or heterodimers in the ground state and excimers or exciplexes in the excited state.^2^ These aggregated species exhibit optical properties distinct from those of their monomeric counterparts. Within the current-to-light or light-to-current conversion^3^ technologies, the important role of dimeric or heterodimeric systems has been demonstrated by numerous studies.^4,5^ For both types of energy conversion, controlling the distance and π–π stacking between monomeric components serves as a powerful tool to modulate molecular and macroscopic properties.

In a π-complex or an exciplex, the vicinity of two molecules serving as the donor and acceptor of electron density enables intermolecular charge transfer (CT) increasing charge separation and usually electron mobility. When applied to organic light-emitting devices (OLEDs), such exciplex CT states can give thermally activated delayed fluorescence (TADF) providing a solution for the triplet harvesting problem in all-organic devices, increasing their external quantum efficiency (EQE) and stability.^6^ On the other hand, in the latest third (TADF) and fourth (hyperfluorescent) generations of OLEDs, the role and potential of dimers or excimers that comprised the same type of monomers remain poorly understood. Apart from the above-mentioned intermolecular CT emitting systems, exciplexes, TADF is more often realized in the covalently bonded donor–acceptor-type emitters. The overwhelming number of such compounds has planar donor and/or acceptor fragments with several conjugated aromatic rings. Such emitters readily show the increase of light emission intensity under precipitation, the so-called aggregation-induced emission, driven by π–π interactions and/or stacking. The latter clearly cannot be neglected in nondoped OLED emissive layers, while in doped films, the effect depends on the emitter and its concentration. For example, archetypal carbazoylcyanobenzenes display intermolecular π–π interactions between carbazole donor fragments giving dimers or aggregates already in the ground state, which strongly affects their TADF properties.^7^ According to the limited reports, aggregation or dimerization of acceptor fragments in a solid state is, however, regarded as a negative phenomenon, causing the decrease of EQE in OLEDs,^8^ generally referred to as excimer quenching.^9^ This is also troublesome for the emitters based on the “multiresonance” effect, including those using DABNA and its analogues as an acceptor in the D–A emitter architecture.^10^ Probably the only exception to this behavior was reported by Chen et al.,^11^ where formation of dimers in a D–A emitter resulted in the improved efficiency and roll-off in the nondoped device.

π–π intermolecular interactions strongly influence electroluminescence color and triplet harvesting,^12^ modulating light emission duration, such as afterglow.^13^ In the current work, we focus on the aggregation effects, which have a potential application in red and near-infrared (NIR) all-organic LEDs. In fact, several recent reports suggest that various cyano derivatives of annulated quinoxaline and phenazine with large planar aromatic systems used as acceptors form dimers or aggregates with strong deep-red and NIR emission.^14−19^ In OLEDs, such emitters enable record red shift of the electroluminescence maximum (λ_EL_) up to 1010 nm^17^ or high maximal EQE (EQ*E*_max_) values.^15^ Notably, in selected cases, J-aggregate formation can significantly reduce singlet–triplet energy gap (Δ*E*_ST_), enhancing TADF and enabling efficient triplet exciton harvesting. This suppression of nonradiative transitions facilitates the development of highly efficient organic emissive materials, including in the NIR region.^14^ Unfortunately, such devices suffer from significant EQE roll-off but also low operational stability even more than OLEDs emitting in the visible range or phosphorescent NIR OLEDs using heavy metal emitters like Pt complexes.^20^

From the applicative point of view, to compete with the second-generation phosphorescent OLEDs, the stability of all-organic TADF and hyperfluorescent analogues needs to be substantially increased. First of all, the EQE roll-off at high current density should be decreased which requires faster radiative deactivation of triplet excitons on the molecular level. One of the main reasons of lower stability of all-organic TADF OLEDs is the slow rate of reverse intersystem crossing (rISC), which describes the transition of “dark” triplet excitons to the emissive singlet ones. Among a few other approaches, introduction of p-block heavy atoms (HAs) like bromine^21^ or selenium^22−24^ has been demonstrated as a promising way to improve the rISC rate and EQE roll-off though the enhancement of spin–orbit coupling (SOC). However, when applied to complex organic emitters, the HA effect remains poorly understood, with often unpredictable structure–property relationships. Previous investigations revealed that (i) random introduction of heavy atoms does not necessarily accelerate rISC,^25^ (ii) direct ISC can be more sensitive to the presence of an HA than rISC,^26^ which is a negative factor for OLED performance, and (iii) the **internal heavy-atom effect** is strongly dependent on the molecular conformation and in some cases is enabled by movements of HAs within specific molecular vibrations. Recently, it has also been reported that in the case of sulfur as a “heavy” atom,^27^ exciplex states between the TADF emitter and a carbazole-based host (mCP) gave a much greater increase in rISC rate than the simple inclusion of the sulfur atom in the TADF molecule. To the best of our knowledge, the nature of the **external heavy-atom effect**(28) and approaches to control it are still unknown.^29^

Regarding the most promising hyperfluorescence approach, which takes advantage of TADF assistant dopants and fluorescent terminal emitters, the design of the external heavy-atom effect for selective and controllable enhancement of rISC is of high interest for high-stability all-organic OLEDs.

**Figure 1:**
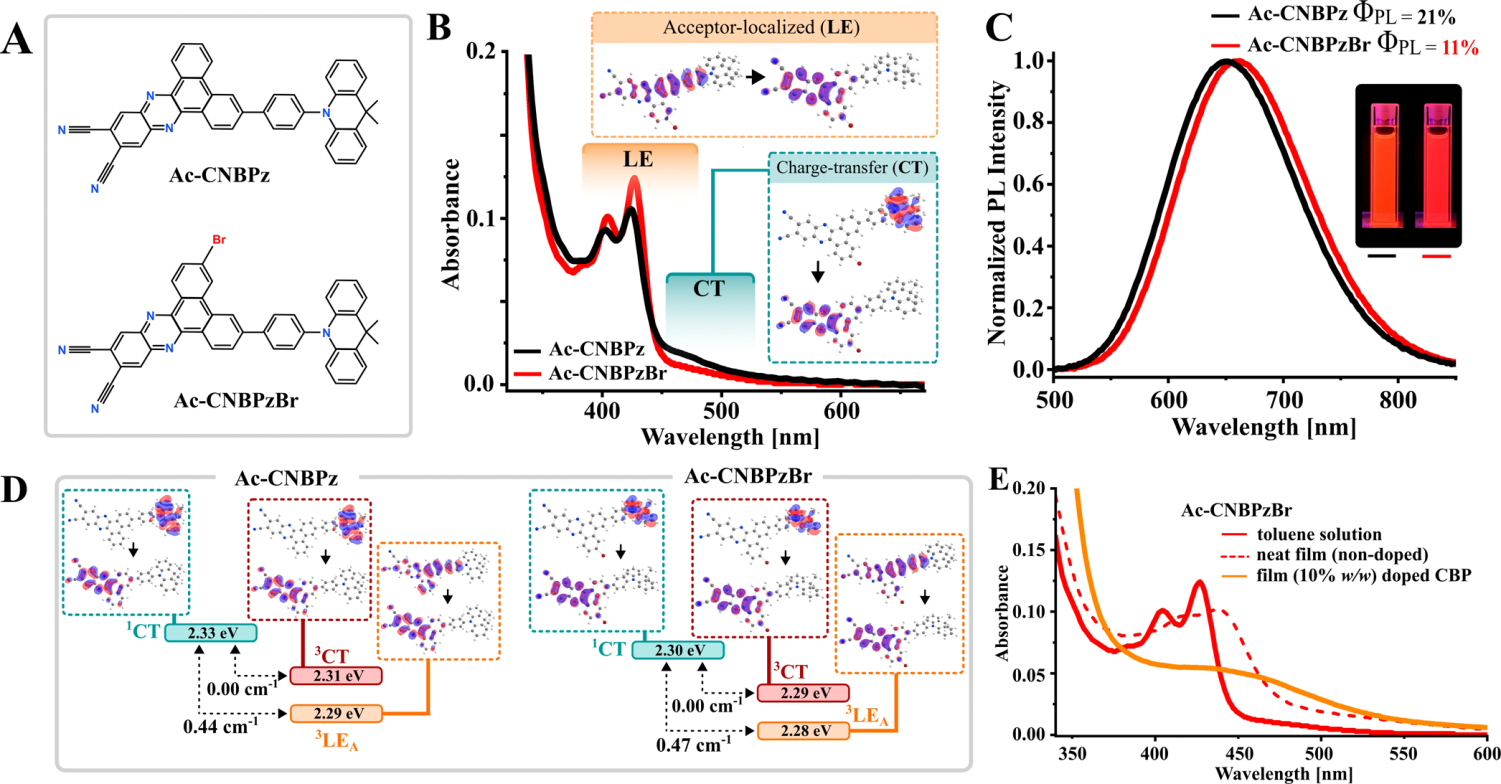
Steady-state properties of investigated emitters. (A) Structures of the investigated emitters, (B) absorption of **Ac-CNBPz** and **Ac-CNBPzBr** in 2 μM toluene solutions; inset: molecular orbitals of the charge-transfer (CT) and locally excited (LE) transitions, (C) photoluminescence (PL) spectra of emitters in the N_2_-purged toluene solutions, (D) energy level diagrams with the molecular orbitals and SOC for respective states, and (E) comparison of the absorption of **Ac-CNBPzBr** in toluene solutions with nondoped and the 10% (*w*/*w*) doped CBP film.

Among other benefits like reduction of energy losses due to restriction of vibrations, application of π–π stacking and dimer or excimer emitters affords self-assembly which can be specifically valuable for the weakly controllable intermolecular effects, like the above-mentioned external heavy-atom effect (EHAE). In this work, we present what we believe is the first report explaining the nature of the external heavy-atom effect in a dimer of a TADF emitter. First, we reveal that **Ac-CNBPz** (Figure 1A) with 9,9-dimethyl-10-phenyl-9,10-dihydroacridine (DMAC-Ph) donor and 11,12-dicyanodibenzo[*a*,*c*]phenazine acceptor forms dimers through stacking of large, planar, π-conjugated acceptor units. When the acceptor is decorated with a pendant bromine atom, direct interaction of the electronic density on the Br atom with the π-electronic density of the dimer is responsible for a SOC enhancement of up to 100-fold. In such a case, SOC is a function of distance and overlap between atomic orbitals of Br and the electronic densities on the very atoms taking part in the electronic spin-flip transition. Under experimental conditions, the presence of such dimers combined with the HA effect results in the enhancement of rISC up to 15-fold and the decrease of EQE roll-off from 4.1% down to 1.1% (@10^2^ cd m^–2^) in a red TADF OLED and from 46% to 32% (@1 mW cm^–2^) in an NIR hyperfluorescent OLED with λ_EL_ = 794 nm.

## Results and Discussion

First of all, we revisited the spectral properties of **Ac-CNBPz**, previously reported as a monomeric TADF emitter^30^ and then compared it with the **Ac-CNBPzBr** derivative bearing a bromine atom in the acceptor fragment (Figure 1A). In dilute 2 μM toluene solutions, where the emitting species are isolated from each other, both emitters are almost identical regarding absorption (Figure 1B) and emission spectra (Figure 1C), emission decays (Figure S1, Supporting Information) as well as lifetimes of prompt and DF, ISC, and rISC rates (Table S1, Supporting Information), and SOC constants (Figure 1D). The vibronically structured phosphorescence spectra measured in frozen methylcyclohexane at 77 K suggest an LE nature of T_1_ (^3^LE) and its energy of 2.28 eV for both emitters (Figure S2, Supporting Information), which is supported by density functional theory (DFT) calculations (Figure 1D) and our previous studies.^31^ The energy gap between the charge-transfer S_1_ (^1^CT) and ^3^LE states is thus estimated to be on the similar level too (Table S2, Supporting Information). Strikingly, the electronic density of Br is not involved in any of the low-energy electronic transitions in **Ac-CNBPzBr**, including the ^3^LE state localized on the acceptor, which explains the same SOC and rates of ISC and rISC for both compounds. Therefore, one can conclude the effective lack of the internal heavy-atom effect in the case of monomeric **Ac-CNBPzBr** species.

**Figure 2:**
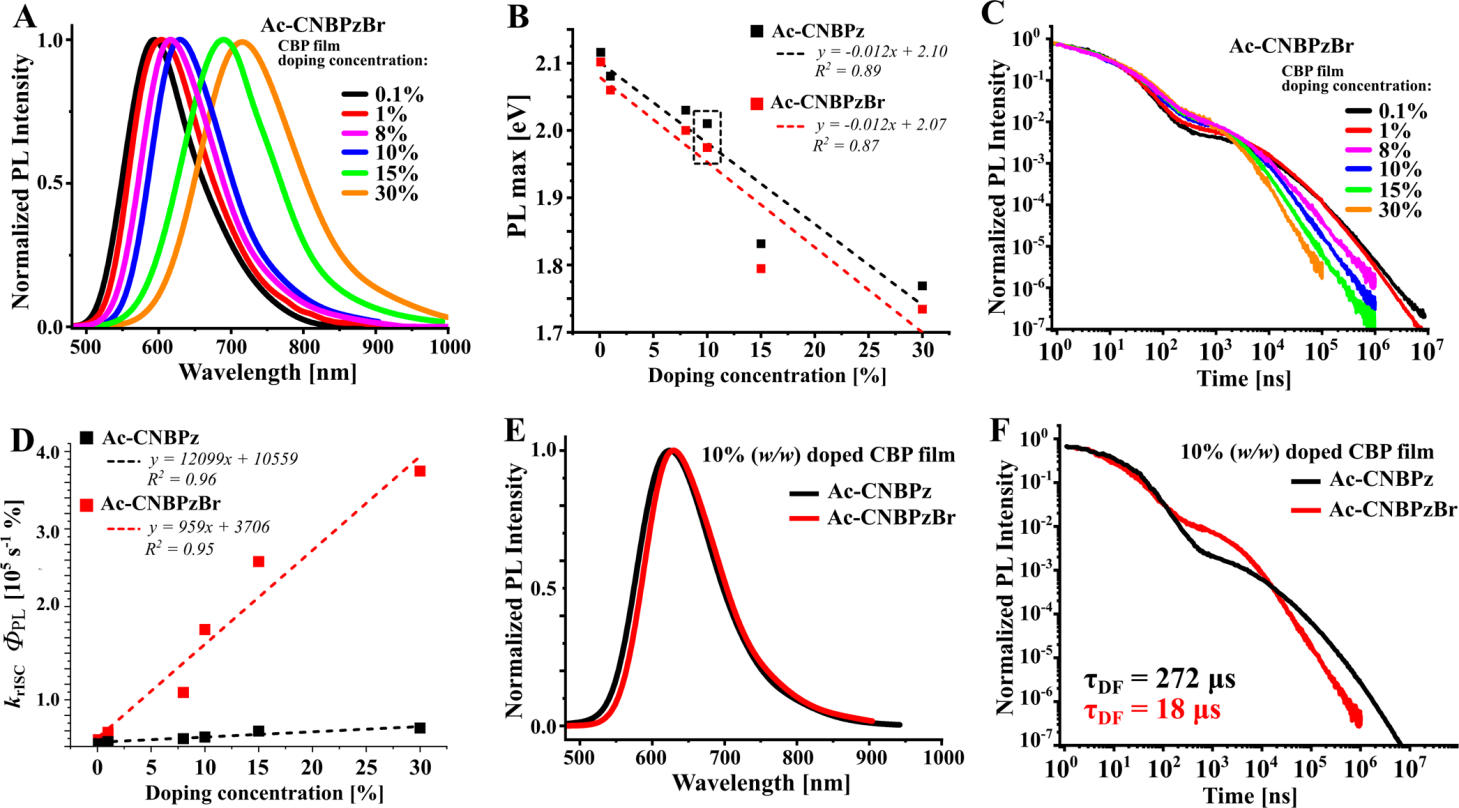
Steady-state and time-resolved emission properties of emitters. (A) Dependency of steady-state PL spectra on concentration in **Ac-CNBPzBr** doped in the CBP film, (B) linear dependency of the PL maximum on doping concentration in both emitters, (C) PL intensity decays of **Ac-CNBPzBr** in CBP films, (D) linear dependency of the product of Φ_PL_ × *k*_rISC_ on doping concentration in both emitters, (E) steady-state PL spectra of **Ac-CNBPz** and **Ac-CNBPzBr** in a 10% doped CBP film, and (F) PL intensity decays of emitters in 10% (*w*/*w*) doped CBP.

The photophysical properties of both emitters were further investigated in films using CBP as a host. In films, the absorption bands of both emitters are red-shifted, indicating formation of aggregates in the ground state (Figures 1E and S3A,B, Supporting Information). Both in neat and doped CBP films, the long-wavelength absorption maximum reaches 445 nm. In doped films, the respective band is, however, much broader, probably due to overlapping absorption of several species. In PL spectra, both emitters show an identical bathochromic shift with the increase of doping concentration, indicating stabilization of the emissive S_1_ state (Figures 2A,B and S4A,B, Supporting Information). **Ac-CNBPz** shows increasing rISC rate (Figure S4C, Supporting Information) with increased doping concentration, followed by the decrease of Φ_PL_ (Table S1, Supporting Information). To eliminate the differences in concentration quenching, the rISC rate constant (*k*_rISC_) is multiplied by Φ_PL_ under vacuum, and this product shows a linear dependence on concentration. In systems similar to **Ac-CNBPz**, such an effect is explained by the decrease of the singlet–triplet energy gap.^12^ A much more pronounced effect is observed in **Ac-CNBPzBr**. According to the slope of the fitted linear *k*_rISC_.Φ_PL_ dependence, the product *k*_rISC_.Φ_PL_ displays a 12-fold larger gradient for **Ac-CNBPzBr** (Figure 2C,D). In the film containing 30% **Ac-CNBPzBr**, the estimated *k*_rISC_ reaches 3.2 × 10^6^ s^–1^, which is almost 30-fold higher than that of **Ac-CNBPz**. Such an enormous effect of concentration clearly indicates the key role of intermolecular interactions in the TADF mechanism and may be an indication for the external heavy-atom effect to be at work. Deactivation rate constants for S_1_, such as *k*_r_ and *k*_ISC_, do not show such strong dependences on either concentration or the presence of the heavy atom (Table S1, Supporting Information).

**Figure 3:**
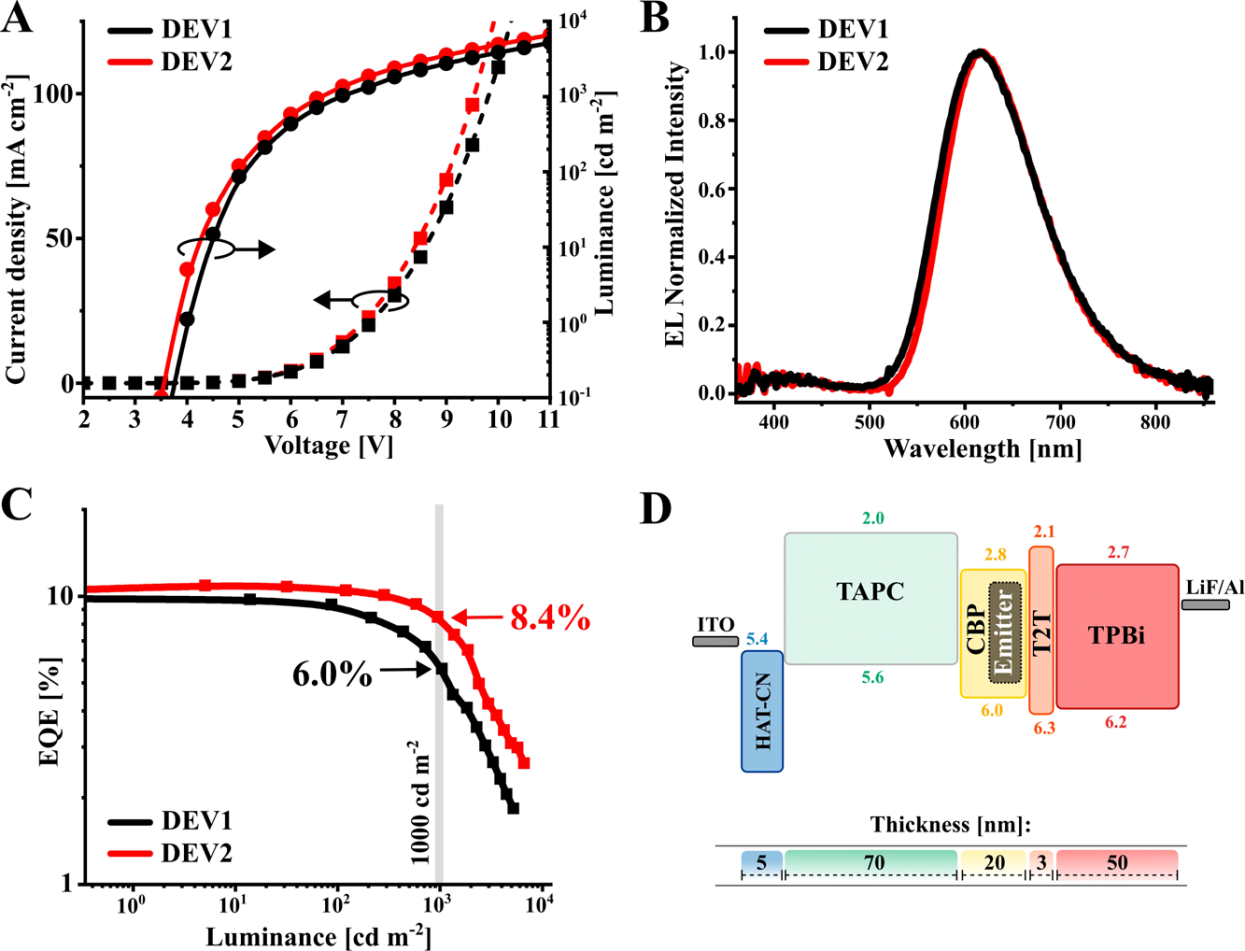
Electroluminescent performance of the TADF OLEDs. (A) *J*–*V*–*L* characteristics, (B) electroluminescence spectra, (C) EQE vs luminance, and (D) structure of the OLED stack used.

We found doping concentrations of ∼8–10% to be optimal and used them for OLED studies (Figure 2E,F). Details of device fabrication and structure (Figure 3D) are shown in the SI, Section S2. In this case, **Ac-CNBPzBr** displays almost a 20-fold higher *k*_rISC_ (*k*_rISC_ = 3.0 × 10^5^ s^–1^) and a 15-fold shorter DF lifetime (τ_DF_) than **Ac-CNBPz**, while also maintaining a satisfactory Φ_PL_ value of 50% (Table S1, Supporting Information). The effect of the heavy-atom-enhanced rISC on electroluminescence was investigated in TADF OLEDs fabricated by vacuum deposition. The device using **Ac-CNBPz** (DEV1) serves as a reference. The device using **Ac-CNBPzBr** as an emitter (DEV2) shows red electroluminescence with an overall slightly higher current density and luminance as compared to DEV1 (Figure 3A,B and Table 1). Importantly, there was a slightly higher EQE_max_ of 10.5% in DEV2 vs 9.7% in DEV1; the brominated emitter affords a substantial decrease in EQE roll-off. At a luminance of 100 cd m^–2^, the EQE roll-off of DEV2 is only 1.1%, nearly 4-fold lower than that for DEV1 (Figure 3C). The roll-off remains better in DEV2 than in DEV1 by a factor of at least two also at higher current densities.

**Table 1:** Electroluminescent Performance of Fabricated OLED Devices

	EML	λ_EL_^a^	*L*_max_^b^	*V*_on_^c^	CE_max_^d^	PE_max_^e^	EQE_max_^f^	EQE/roll-off^g^	
		[nm]	[cd m^–2^]	[V]	[cd A^–1^]	[lm W^1–^]		@10^2^ cd m^–2^	@10^3^ cd m^–2^
DEV1	**CBP:Ac-CNBPz** (92:8)	614	5330	3.5	13.1	10.9	9.7%/---	9.3%/4.1%	6.0%/38.1%
DEV2	**CBP:Ac-CNBPzBr** (92:8)	619	6750	3.5	14.8	11.5	10.5%/---	10.4%/1.1%	8.4%/20.1%

	EML components	λ_EL_	*R*_max_^h^	*V*_on_	CE_max_	PE_max_	EQE_max_	EQE_NIR_/roll-off		
		[nm]	[mW cm^–2^]	[V]	[cd A^–1^]	[lm W^1–^]		EQE_max(NIR)_^i^	@1 mW cm^–2^	@3 mW cm^–2^
DEV3	**CBP:Ac-CNBPz:BPPC** (91.25:8:0.75)	794, 624	5.9	3.5	6.7	5.2	6.1%/---	3.4%/---	1.8%/47%	0.9%/74%
DEV4	**CBP:Ac-CNBPzBr:BPPC** (91.25:8:0.75)	794, 623	5.4	3.5	7.1	5.5	7.2%/---	4.1%/---	2.8%/32%	1.9%/54%
DEV5	**CBP:Ac-CNBPzBr:BPPC** (78.25:20:1.75)	798	1.3	3.5	0.8	0.6	0.75%/---	0.72%/---	0.45%/39%	–/–

a Electroluminescence maxima.

b PL maxima.

c Turn-on voltage.

d Maximum value of current efficiency.

e Maximum value of power efficiency.

f Maximum EQE.

g Roll-off was calculated as 100% × (EQE – EQE_max_)/EQE_max_.

h Radiant emittance.

i EQE_max(NIR)_ is EQE_max_ calculated for NIR wavelength range (700–1000 nm).

**Figure 4:**
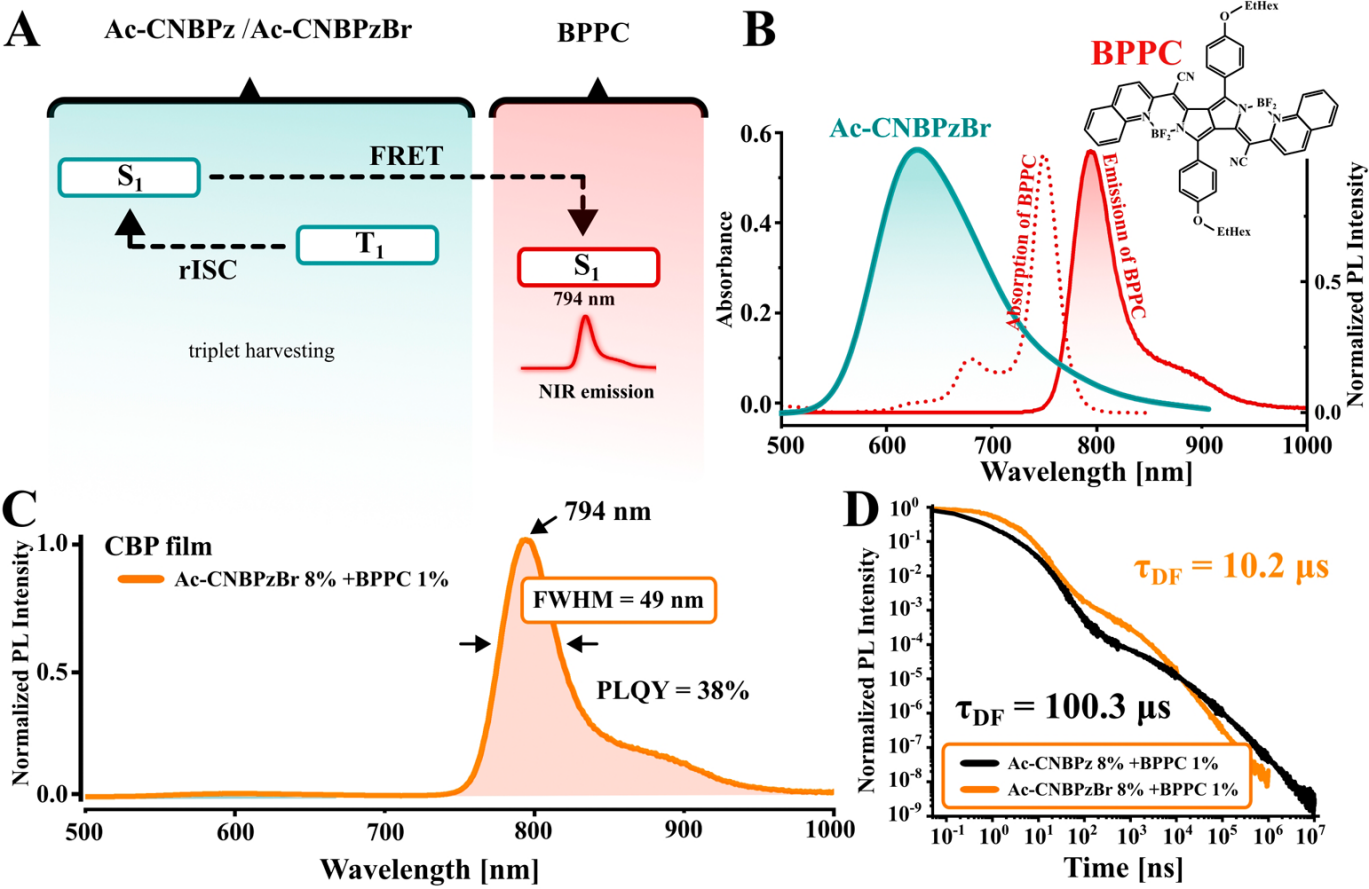
Photophysical properties of the TADF-sensitized NIR system consisting of the TADF emitter combined with the **BPPC** fluorophore. (A) Suggested mechanism of hyperfluorescence; (B) PL spectrum of **Ac-CNBPzBr** (FRET donor) and absorption and PL spectra of **BPPC** (FRET acceptor); (C) PL spectrum of the film containing **BPPC** (1%) and **Ac-CNBPzBr** (8%) in a CBP host; and (D) normalized PL intensity decays of the hyperfluorescent systems.

We also studied the hyperfluorescence^32,33^ approach to see whether the improved triplet-harvesting properties of **Ac-CNBPzBr** can be used for NIR electroluminescence (Figure 4A). To achieve this, we used the NIR fluorescent dye **BPPC**(34) (Figure 4B) with a maximum λ_PL_xat 794 nm as a terminal emitter, while **Ac-CNBPz** and **Ac-CNBPzBr** served as assistant dopants. In PL experiments, the **CBP**:**Ac-CNBPzBr**:**BPPC** blend (91:8:1, *w*/*w*) shows pure NIR emission with the Φ_PL_ of 38% (Figure 4C) and the τ_DF_ = 10.2 μs (Figure 4D), an order of magnitude smaller than that when **Ac-CNBPz** is used as an assistant dopant (τ_DF_ = 100.3 μs).

**Figure 5:**
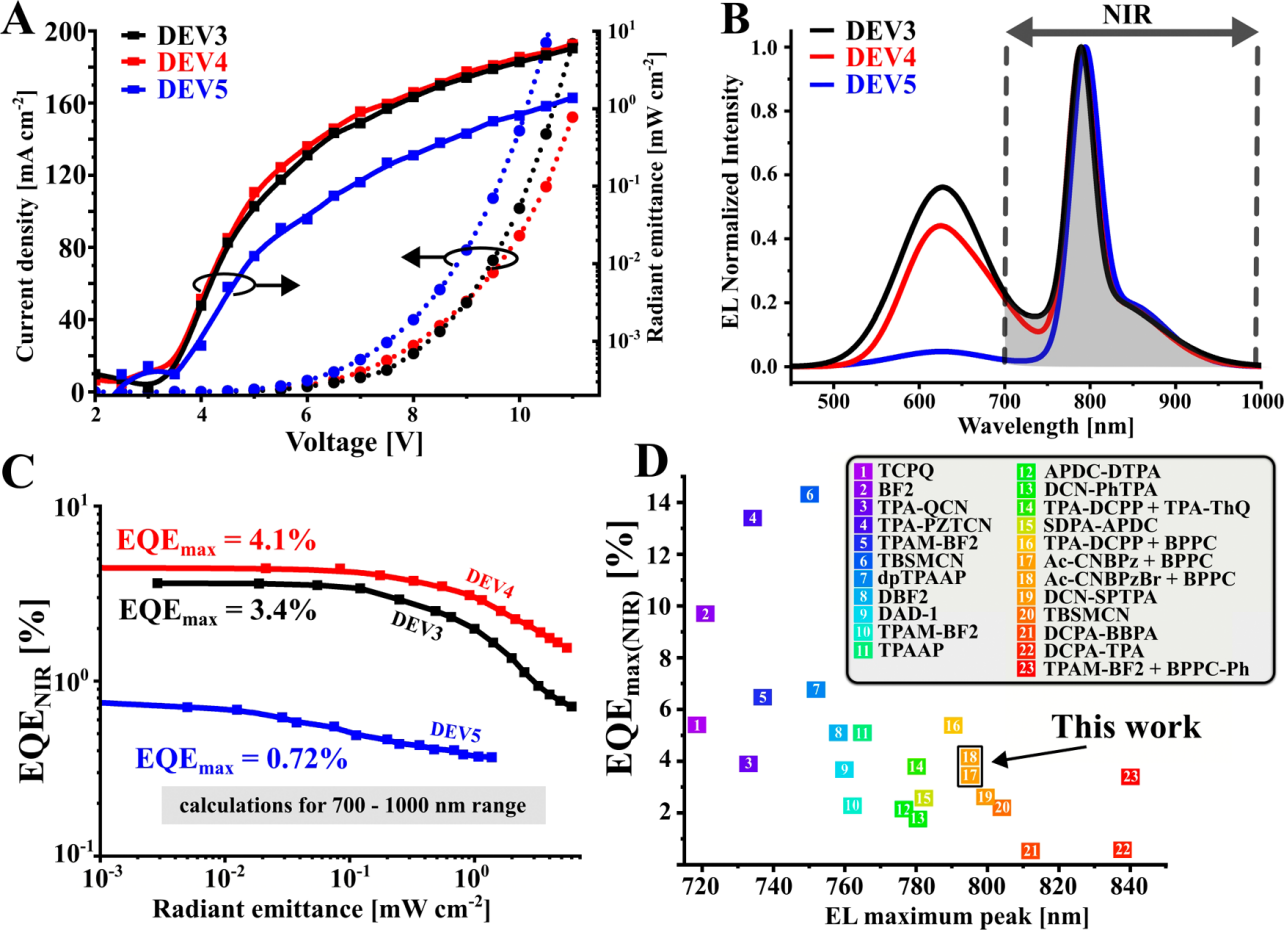
Electroluminescent performance of HF-based OLEDs. (A) *J*–*V*-radiant emittance characteristics of DEV3–5 devices, (B) electroluminescence spectra, (C) EQE vs radiant emittance plot (EQE_NIR_ is calculated for the 700–1000 nm range), and (D) comparison of the EQE_max_ of the state-of-the-art all-organic NIR OLEDs emitting in the 715–840 nm range; for the references, see the Supporting Information.

The fabricated HF OLEDs had a similar structure as the TADF counterparts (Figure 4D and Supporting Information, Section 2). Their electroluminescent parameters are summarized in Table 1 and Figure 5A–C. The combination of **Ac-CNBPzBr** and **BPPC** in CBP in the emissive layer of device DEV4 results in a 20% higher EQE_max_ of 7.2% and almost 1.5 lower roll-off compared to the device DEV3 with **Ac-CNBPz** as an assistant dopant (Table 1). Although we observed a nearly 99% efficiency of FRET from **Ac-CNBPz/Ac-CNBPzBr** to **BPPC** in photophysical experiments, the electroluminescence spectra display a considerable amount of emission originating from the assistant dopant. This indicates that some of the excitation of the assistant dopant is not transferred to **BPPC**, and the **Ac-CNBPz/Ac-CNBPzBr** emission rate outcompetes FRET at low terminal emitter concentration.^35^ It can be attributed to the different preparation of samples for photo- and electroluminescent experiments. Much higher NIR purity is achieved in the DEV5 device with increased concentrations of both **Ac-CNBPzBr** to 20% and **BPPC** to 1.75%, although this device displays a lower EQE. As compared to the state-of-the-art all-organic NIR OLEDs^18,33,36^ (Table S3, Supporting Information), in terms of EQE_max_, the performance of the DEV4 device is record high. As for the NIR range, the EQE_max(NIR)_ of 4.1% is somewhat lower as compared to 5.4% reported previously for a similar HF device with **TPA-DCPP** as an assistant dopant^34^ (Figure 5D). However, the latter OLED suffers from a strong roll-off exceeding 67% at 10 mA cm^–2^, while in DEV4, the respective value is almost three times lower: 25%.

To understand the origin of the heavy-atom effect causing faster rISC and improvement of the EQE roll-off in the OLEDs, the photophysics of emitters in the 10%-doped films was studied in detail. Both emitters showed a similar red shift in the prompt fluorescence (PF) region and a blue shift in the DF region (Figure S5A–C, Supporting Information), evidencing a similar distribution of dihedral angle between the donor and acceptor. Surprisingly, even at 10 K, the emitters maintain similar spectral behavior as at room temperature with only 2 orders of magnitude decrease in the intensity of DF amplitude (Figure S6A, Supporting Information). Almost identical PF and DF spectra at 10 K (Figure S6B, Supporting Information) most likely indicate a negligible energy gap Δ*E*_ST_ in both emitters in CBP. The described similarity of spectral behaviors of **Ac-CNBPz** and **Ac-CNBPzBr** indicates that the significant rISC enhancement cannot be explained exclusively by the differences in Δ*E*_ST_ (see Table S4, Supporting Information).

**Figure 6:**
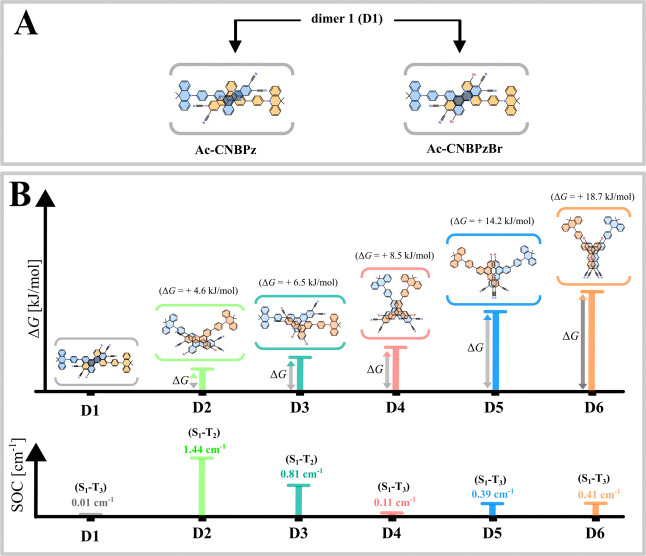
Various dimers of the investigated emitters. (A) Schematic representation of the crystal phase structure of the D1 dimers. (B) Schematic representation of various dimers of **Ac-CNBPzBr** in their ground-state optimal geometries with the Gibbs free energy of formation (Δ*G*) and SOC constants for the lowermost S_n_–T_n_ transitions with a nonzero SOC constant. Note that the D1 structure lacks offset in optimal geometry under vacuum and is different from that in the crystal phase. Optimization was performed using the M062X/LANL2DZ method.

The above-mentioned large effect of concentration on rISC in **Ac-CNBPz** and especially **Ac-CNBPzBr** indicates strong influence of aggregates. In fact, in the crystal phase measured by X-ray diffraction (XRD),^30^**Ac-CNBPz** forms dimers (D1) in which the monomer molecules are arranged in a face-to-face alignment and linked by an offset π–π stacking interaction. We also undertook XRD analysis of **Ac-CNBPzBr** crystals, revealing the same type of D1 dimers (Figures 6A and S7A–D, Supporting Information). Only in the case of **Ac-CNBPzBr** are monomer molecules aligned without any offset. It should be noted that single crystals used in the XRD analysis could only be obtained from 1,4-dioxane (Figure S8, Supporting Information), which is present in the crystal structure in a 1:1 ratio with **Ac-CNBPz** and **Ac-CNBPzBr**. None of these crystals are fluorescent, which is analyzed in detail in Section 4, including Figures S9 and S10, Supporting Information.

Importantly, the above-described heavy-atom effect on TADF and the performance of the OLED was observed for amorphous films but not crystals, and those did not contain 1,4-dioxane. Unfortunately, numerous attempts to obtain single crystals from other solvents were unsuccessful; both compounds form amorphous precipitates instead of crystallization. We assume that under such conditions, aggregation is driven by various kinetic factors, especially strong π–π stacking, resulting in less regular dimerization and/or formation of species of different kind(s). In fact, DFT calculations predict that several kinds of π–π stacking dimers have close energies of formation (Figures 6B and S11, Supporting Information).

In **Ac-CNBPz**, the formation of dimers does not induce SOC enhancement. According to the Gibbs free energies of formation (Δ*G*), dimers D2 and D3 in Figure S11 (Supporting Information) can accompany dimer D1 to a certain extent, but maximal SOC remains below 0.1 cm^–1^. The SOC of dimer D4 is comparable with that of the **Ac-CNBPz** monomer; however, formation of D4 is not favored thermodynamically. Such analysis suggests that in **Ac-CNBPz**, the aggregation effects do not enhance spin-flip transitions, which is in fact the case observed in the experiment (Figure 2E).

**Figure 7:**
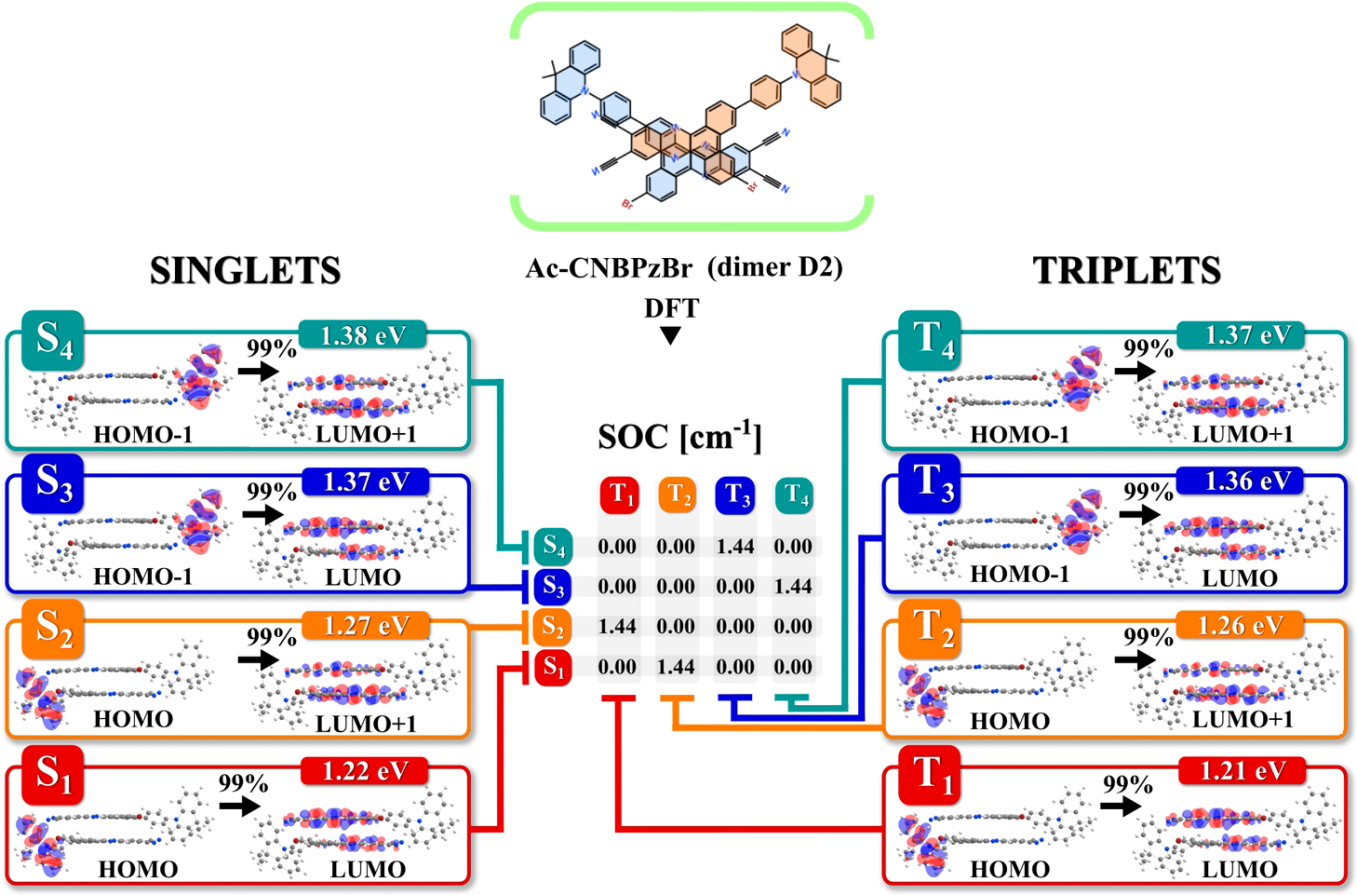
Geometry of the dimer D2 of **Ac-CNBPzBr** optimized for the ground state, molecular orbitals forming the lowest excited states, and respective SOC constants. Calculations of excited-state properties were performed on the B3LYP/LANL2DZ level of theory.

**Figure 8:**
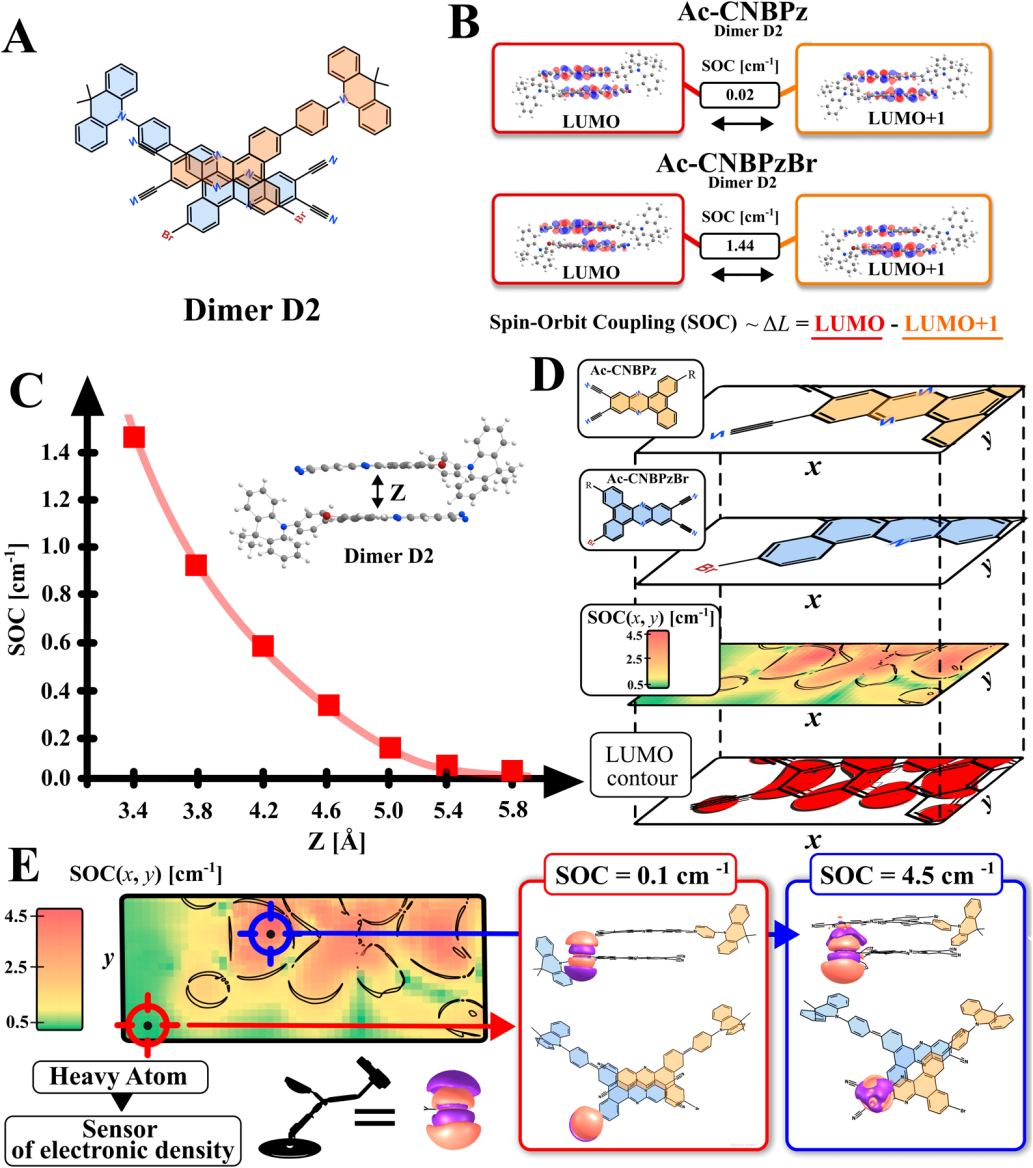
Detailed analysis of the dimer D2. (A) Structure of dimer D2 of **Ac-CNBPzBr**; (B) change of the angular momentum (Δ*L*) and SOC constants of the S_1_–T_2_, S_2_–T_1_, S_3_–T_4_, and S_4_–T_3_ transitions in D2 of **Ac-CNBPz** and **Ac-CNBPzBr**; (C) dependency of SOC constant for the S_1_–T_2_ transition on the relative distance Z between monomers in dimer D2 of **Ac-CNBPzBr**; and (D) fragment of the two-dimensional (*x*, *y*) map of the S_1_–T_2_ transition SOC constant of a heterogenic **Ac-CNBPz**–**Ac-CNBPzBr** dimer D2; the LUMO contour fragment is shown for comparison. (E) Two extreme positions of the 4p atomic orbital of Br: with and without an overlap with the π-electronic density of the neighboring monomer molecule and respective max. and min. SOC positions on the diagram in Figure 8D.

On the other hand, in **Ac-CNBPzBr**, all of the analyzed dimers D2-D6 show much higher SOC as compared to D1 found in crystals (with 1,4-dioxane), while in D2 and D3, it substantially exceeds the SOC of the monomer. Dimer D2 with face-to-face configuration and C2 symmetry (Figures 7 and 8A) is of specific interest due to its low Δ*G* and high SOC reaching 1.44 cm^–1^ in the optimized geometry. In fact, the appearance of even some portion of D2 in films could explain the increased rISC of **Ac-CNBPzBr** and decreased roll-off in the OLEDs. Such a relatively high SOC value is predicted for four electronic transitions, namely, S_1_–T_2_, S_2_–T_1_, S_3_–T_4_, and S_4_–T_3_, which involve the distribution of electronic density within two acceptor fragments of the dimer D2 (Figure 8B). The resulting change of orbital angular momentum (Δ*L*), described by lowest unoccupied molecular orbital (LUMO) and LUMO + 1 (Figure 8B), is considerable enough to enable a minimal SOC of 0.02 cm^–1^ in the same D2 dimer type of **Ac-CNBPz** (Figure S12, Supporting Information). Taking into account almost identical Δ*L* in both emitters (Figure 8B), we believe it is the presence of bromine atom(s) in D2 of **Ac-CNBPzBr** which causes such a large increase in SOC by more than 70 times. Above mentioned refers to the lowest excited states of intermolecular charge-transfer character. In terms of higher SOC, LE triplet states in D2 of **Ac-CNBPzBr**, as well as of **Ac-CNBPz**, are unlikely to facilitate rISC substantially due to lower SOC values and high energy of ^3^LE as predicted by DFT calculations (Figure S13A,B, Supporting Information).

Since the electronic density on the bromine atoms is not involved in any low-energy elctronic transitions of D2, its effect cannot be regarded as internal. The same is true for the monomeric species of **Ac-CNBPzBr**, for which the lack of a heavy-atom effect was concluded above. On the other hand, the computational results support the observed experimental evidence on the external nature of the heavy-atom effect. Namely, SOC was found to be sensitive to the mutual arrangement of the two acceptor fragments. First of all, the distance between the two monomers is an important factor, as is depicted in Figure 8C. Namely, SOC grows exponentially with the decrease of the distance between the bromine atom of one molecule and the π-electronic density of the second acceptor involved in the electronic transition. To further investigate this, we performed a simplified computational experiment: a heterogenic D2-type dimer of **Ac-CNBPzBr** and **Ac-CNBPz** was analyzed to eliminate the possible synergic effect of two bromine atoms. The brominated monomer was scanned within the XY acceptor plane of **Ac-CNBPz** (Figure 8D) keeping the distance between monomers fixed. The resulting 3D map of SOC vs XY coordinates of the **Ac-CNBPz** acceptor plane shows the maximal SOCMEs for the closest positions of Br and respective C atoms involved in the electronic spin-flip transitions. Respectively, SOC minima are observed when Br is out of the plane of the neighboring acceptor or near the C atoms not participating in the transition. In fact, the projection of such a map perfectly reflects the LUMO orbital of the D2 dimer (Figure 8D). As depicted in Figure 8E, under the most favorable alignment, direct overlap of the 4p atomic orbital of the bromine atom and 2p orbitals of the carbon and nitrogen atoms forming the π-electronic density of neighboring acceptor fragment is the reason of maximal SOC. On the other hand, when such a 4p-π orbital overlap is reduced or absent due to the change of relative position of Br, SOC decreases sharply. Such analyses indicate that the direct overlap of atomic orbitals of the external HA with the π-electronic density involved in the triplet–singlet transitions is the key to maximizing the rISC rate. The same simulations conducted for the D2 dimer of **Ac-CNBPzBr** indicate that maximal SOC can reach 4.5 cm^–1^. Taking into account mutual movements of monomers in a film at room temperature, the average SOC should oscillate from 0.1 to 4.5 cm^–1^.

**Figure 9:**
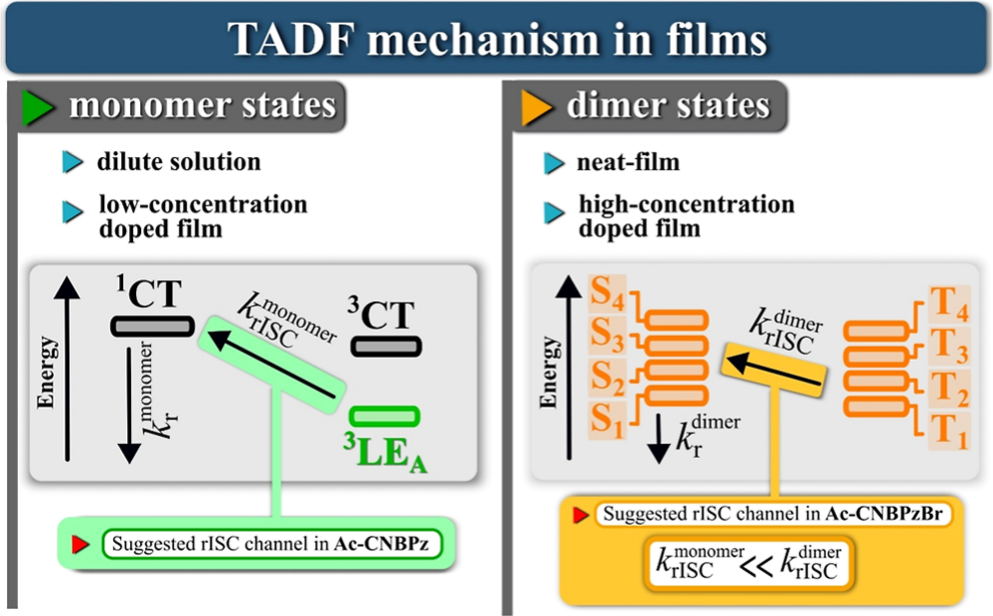
Suggested TADF mechanisms.

Finally, the TADF mechanism in films can be proposed as follows. Various dimers that are formed in the ground state are responsible for the long-wavelength absorption and emission. In the case of **Ac-CNBPz**, SOC within the dimer excited states cannot compete with the ^3^LE-^1^ CT channel of the monomer. Taking into account that in **Ac-CNBPz**, the rISC rate does not decrease with the increasing doping concentration, we speculate it occurs via the monomer excited states which under the conditions of aggregation have higher energy than the dimers (Figure 9). It is also likely that free monomer species of **Ac-CNBPz** undergo TADF, while the coexisting dimer or aggregates are TADF inactive. The presence of bromine in the vicinity of the electronic density of the LUMO of dimer activates a much more efficient rISC channel involving the dimer excited states. Not only the energy gap is smaller for such a transition but also SOC is immensely affected by the external heavy-atom effect. The combination of these factors provides superior triplet harvesting properties of **Ac-CNBPzBr** in the films. We should note that due to the possible coexistence of monomers and various dimers showing similar spectral behavior, the TADF mechanism in studied macroscopic systems can be much more complex and involve several aggregates.

## Conclusions

In the context of the fast development of organic optoelectronics, it is important to understand and control the intermolecular factors that define the (electro)luminescent properties of molecular materials. According to the XRD analysis, concentration-dependent absorption and photoluminescence measurements, the π–π stacking-driven homotypic interactions of the emitter molecules in films are responsible for the EHAE on TADF. In our example, an **Ac-CNBPz** dimer system displays near-zero SOC between the lowest excited states, while the molecule appended with a Br atom shows an incredible boost of SOC constants up to 200-fold. To maximize this effect, in a specified dimer, the local electronic density of the HA should interact directly with the electronic density of the atom(s) involved in the electronic transitions, as SOC shows an exponential dependence on the respective distance. Achieving a direct orbital overlap between the heavy-atom orbitals and electronic transition density is thus the key to maximize the rISC. Regarding such an external heavy-atom effect, the aggregation driven by π–π stacking plays a dual role. On the one hand, it enables a certain extent of self-assembly of the emitter species and affords dimer(s) with favorable rISC parameters. On the other hand, strong π–π stacking decreases the selectivity of aggregation and increases the heterogeneity of the emitting species. As a result, various types of dimers and likely other aggregates are present. While having a minor effect on the absorption or emission spectra, this has strong influence on the average SOC and thus triplet harvesting of the final material.

Regarding the EHAE mechanism, we reveal that SOC in such dimers is extremely sensitive to the 4p orbital overlap with the π-electronic density of the second acceptor involved in the electronic transition. Thus, the distance between specifically oriented monomers can be regarded as the key factor affecting this 4p-π interaction and the EHAE in general.

The resulting light-emitting materials containing a brominated emitter **Ac-CNBPzBr** show superior triplet harvesting properties in the red and NIR range. While maintaining the Φ_PL_ value above 50%, the presence of bromine affords a 15-fold shorter lifetime of DF, which results in a 4–2-fold lower EQE roll-off in the TADF OLED. **Ac-CNBPzBr** used as an assistant dopant affords a high EQE of 4.1% in the NIR region, with λ_EL_ 794 nm in the hyperfluorescent OLED. The revealed nature of the external heavy-atom effect opens new possibilities in the design and improvement of organic emitters.

## Methods

### Materials

Reagents for synthesis, solvents of respective grades, and 4,4′-bis(*N*-carbazolyl)-1,10-biphenyl (CBP) for photoluminescence spectroscopy were purchased and used without further purification.

### Synthesis

**BPPC**(34) and **Ac-CNBPz**(30) were synthesized as described previously. For detailed synthetic procedure of **Ac-CNBPzBr**, see Scheme S1, Supporting Information, as well as analyses (Figures S14–S16, Supporting Information).

### Sample Preparation for Photophysical Measurements

Films were prepared on quartz glass by a solution-processing technique using a spin-coating method.

### UV–Vis and Photoluminescence Measurements

UV–vis absorption spectra were recorded by using a Shimadzu UV-1900 spectrophotometer. Steady-state photoluminescence (PL) measurements were conducted using an FS5 spectrofluorometer (Edinburgh Instruments) using front-face excitation geometry with a 1 nm spectral resolution. Absolute PL quantum yields (Φ_PL_) were measured using an integrating sphere, included in the FS5 spectrofluorometer. Time-resolved measurements were performed using a customized system^37^ consisting of a pulsed YAG:Nd laser (PL2251A, EKSPLA) coupled with an optical parametric generator (PG 401/SH) as the excitation light source and 2501S grating spectrometer (Bruker Optics) combined with a streak camera system (C4334-01 Hamamatsu) as the detection unit. The system was equipped with a double-stage high-vacuum pump (T-Station 85 Edwards). To reduce scattering, reflections, and secondary order artifacts, a set of various high-performance optical bandpass (BP) and long-pass (LP) filters were used, in the excitation path 325/50BP, together with an LP filter 375LP (Edmund Optics). To build PL intensity decay profiles, streak camera images were integrated over a constant specified wavelength interval. Phosphorescence measurements were recorded using a closed-cycle helium cryostat (APD DE-202) and a temperature controller (LakeShore 336). Photophysical constant rates *k*_r_, *k*_ISC_, and *k*_rISC_ were calculated according to equations described in the Supporting Information.

### Quantum Chemical Calculations

Quantum chemical calculations were conducted at the DFT/TD-DFT level of theory using the Gaussian 16 program package.^38^ Unconstrained geometry optimizations were conducted using B3LYP^39^ and M06-2X^40^ functionals for monomers and dimers, respectively. Electronic parameters and molecular orbitals in the ground and excited states were calculated using the B3LYP functional. The LANL2DZ basis set was used in the above-mentioned types of calculations. SOC constants were computed using the ORCA 4.2 software package^41^ with the B3LYP functional and the DEF2-TZVP basis set with included relativistic zero-order regular approximation. The SOC values for dimers were calculated with the ground-state geometries.

### OLEDs

For the details of the fabrication of OLEDs, see the Supporting Information.

### Single-Crystal XRD

For the details of XRD experiments, see the Supporting Information.

## Supporting Information Available

Experimental photophysical parameters and detailed procedure of their determination, OLED performance and their fabrication, details of XRD experiments, quantum chemical calculations, and NMR and matrix-assisted laser desorption ionization time-of-flight spectra of emitters and their synthetic procedures (PDF)
