# Supplementary material for: Heavy Atom as a Molecular Sensor of Electronic Density: The Advanced Dimer-Type Light-Emitting System for NIR Emission
Source: ACS Appl Mater Interfaces. 2025 Jan 31;17(6):9635–45. doi: 10.1021/acsami.4c21674 (PMC11826893; doi:10.1021/acsami.4c21674)
Supplement: Supplementary file 1 — am4c21674_si_001.pdf [file am4c21674_si_001.pdf]

Supporting Information

Heavy atom as a molecular sensor of electronic density: the advanced dimer-type light emitting system for NIR emission

Michał Mońka,[1]* Piotr Pander,[2,3] Daria Grzywacz,[4] Artur Sikorski,[4] Radosław Rogowski,[1]

Piotr Bojarski,[1] Andrew P. Monkman,[5] Illia E. Serdiuk[1]*

[1] Faculty of Mathematics, Physics and Informatics, University of Gdańsk, Wita Stwosza 57, 80-308 Gdańsk, Poland

[2] Faculty of Chemistry, Silesian University of Technology, M. Strzody 9, 44-100 Gliwice, Poland

[3] Centre for Organic and Nanohybrid Electronics, Silesian University of Technology, Konarskiego 22B, 44-100 Gliwice, Poland

[4] Faculty of Chemistry, University of Gdansk, Wita Stwosza 63, 80-308 Gdańsk, Poland

[5] Department of Physics, Durham University, South Road, Durham, UK

*Corresponding authors. E-mail: illia.serdiuk@ug.edu.pl, michal.monka@ug.edu.pl, phone + 48 58 523 22 44

TABLE OF CONTENTS

Section S1. Photophysical measurements

Section S2. OLEDs

Section S3. X-ray

Section S4. Quantum chemical calculations

Section S5. NMR, MALDI-TOF spectra of emitters and synthetic procedures

Section S1. Photophysical measurements

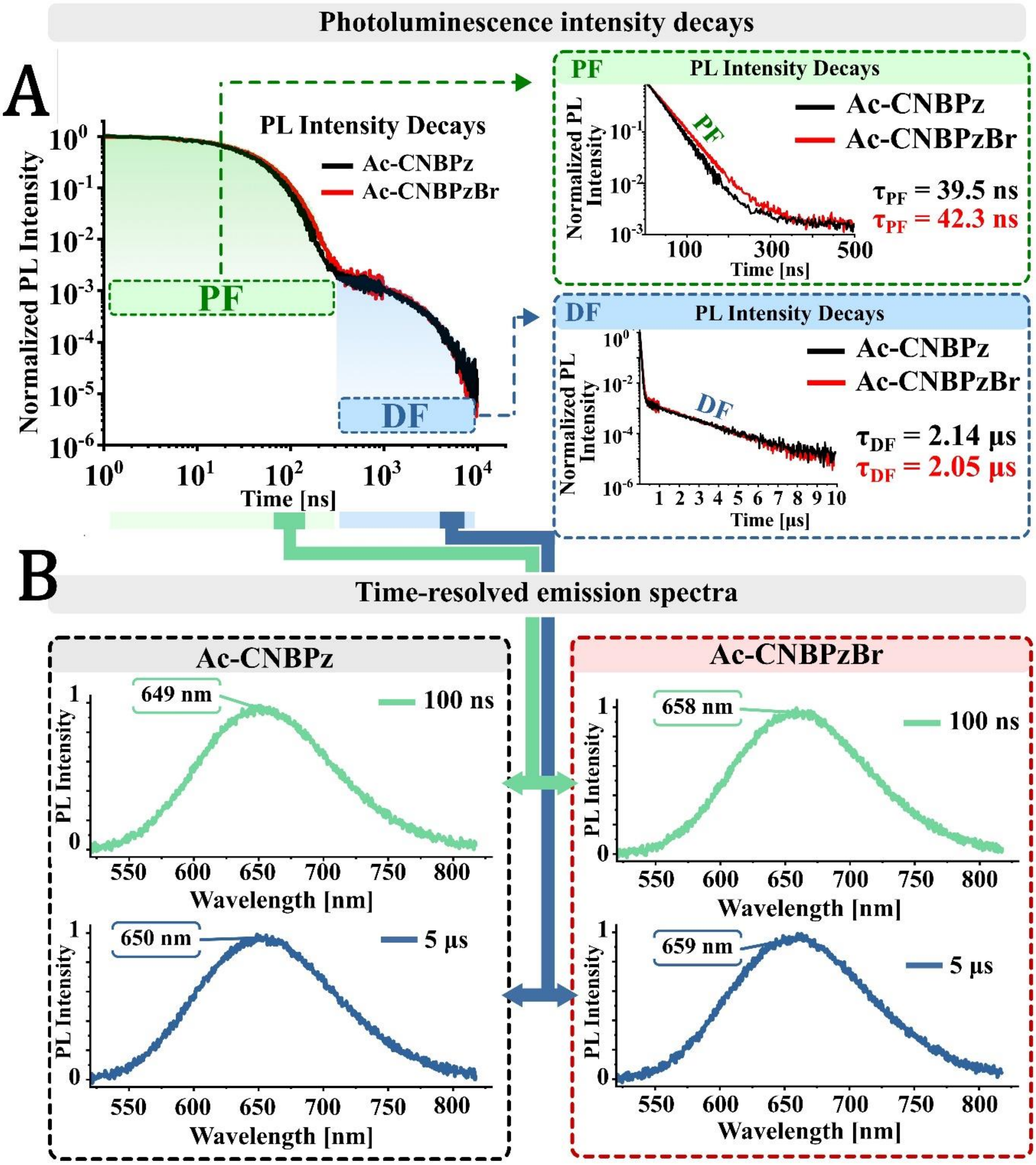

Figure S1. Time-resolved photophysical results of **Ac-CNBPz** and **Ac-CNBPzBr** in N_2-purged toluene solutions. **A**: PL intensity decays, **B**: Time-resolved emission spectra taken at different time delays using streak camera.

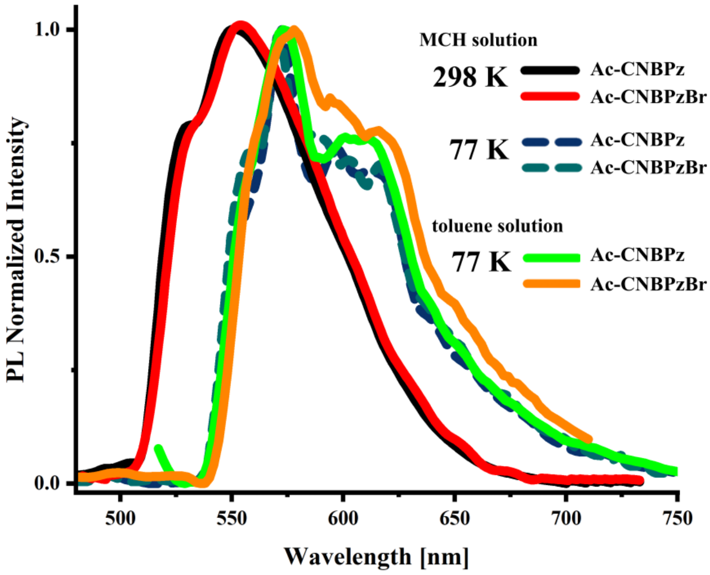

Figure S2. Steady-state photoluminescence and phosphorescence spectra of the studied emitters measured in frozen methylcyclohexane (MCH) and toluene at 77 K and 298 K.

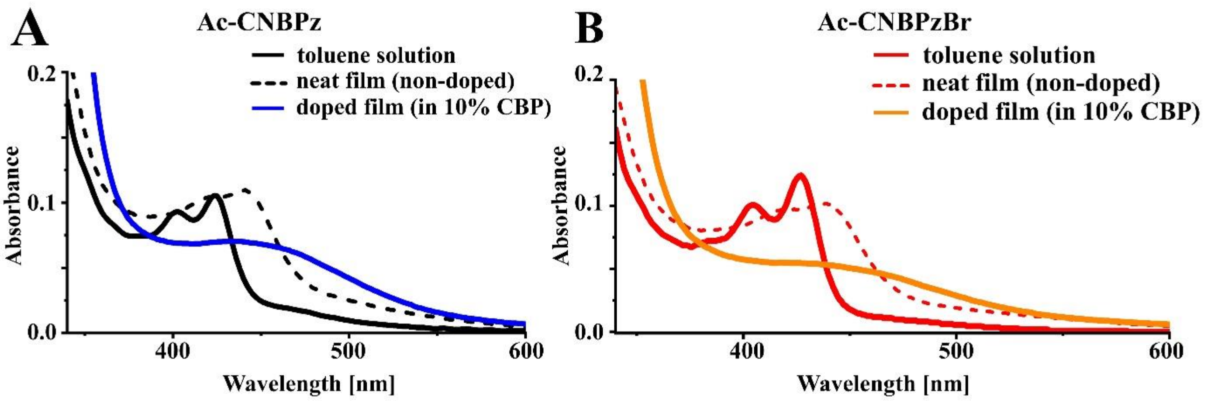

Figure S3. The comparison of the absorption spectra of **Ac-CNBPz** (**A**) and **Ac-CNBPzBr** (**B**) in toluene solution, non-doped and 10% CBP doped films.

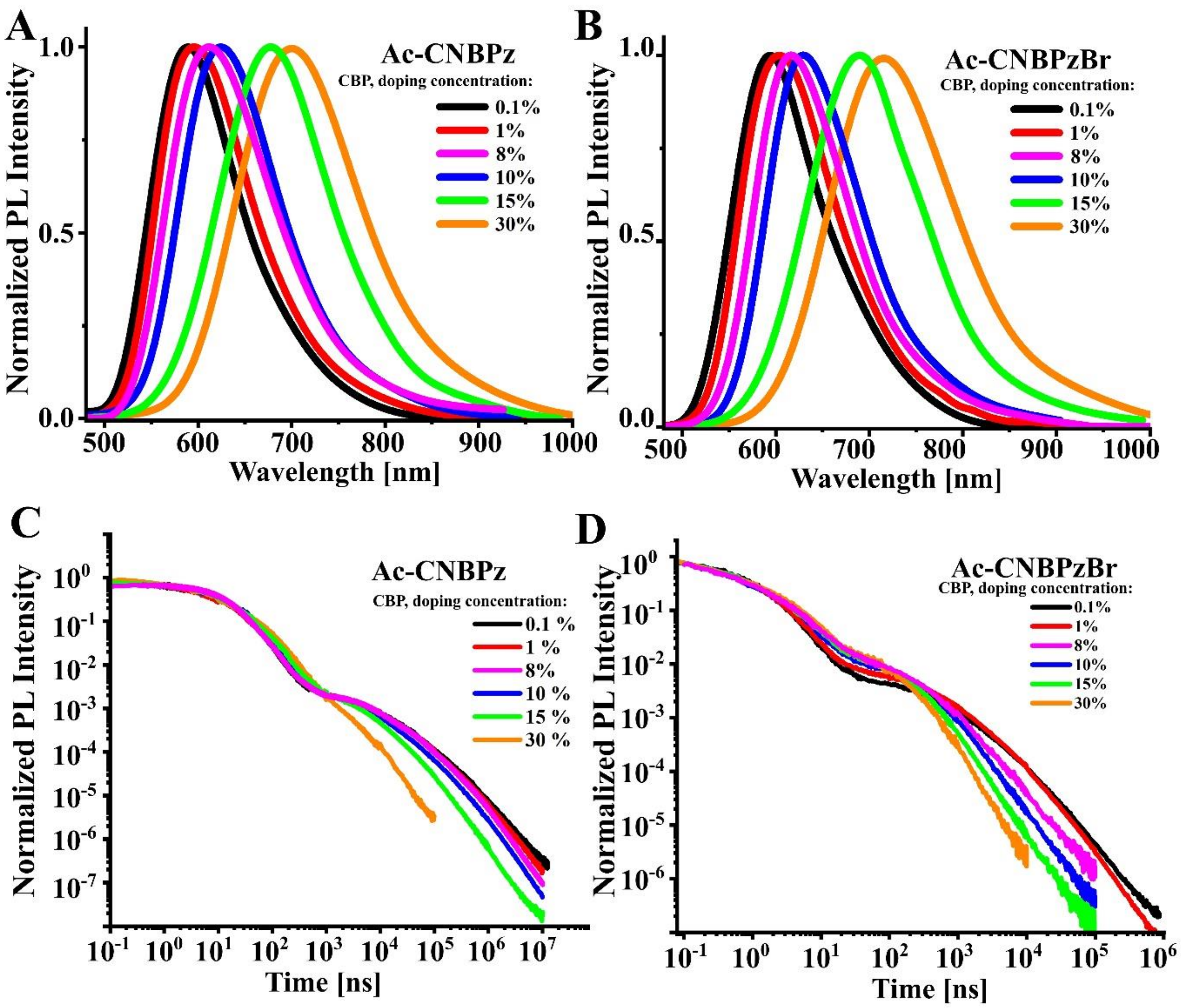

Figure S4. Steady-state PL spectra of **Ac-CNBPz** (**A**) and **Ac-CNBPZBr** (**B**) in CBP doped films in various concentrations, PL Intensity decays of **Ac-CNBPz** (**C**) and **Ac-CNBPZBr** (**D**) in CBP doped films in various concentrations.

Table S1. Photophysical parameters of the studied emitters investigated in toluene solution and CBP doped films.

		Φ_{PL}[a]	Φ_{PL_air}[a]	λ_{PL}[b]	τ_{PF}[c]	τ_{DF}[c]	k_r[c]	k_{ISC}[c]	k_{rISC}[c]
		[%]	[%]	[nm]	[ns]	[μs]	[10^7 s^{-1}]	[10^7 s^{-1}]	[10^4 s^{-1}]
Solutions:									
**Ac-CNBPz**	Tol	21	21	649	39.5	2.14	0.48	0.25	51.8
**Ac-CNBPzBr**		11	11	658	42.3	2.05	0.24	0.21	52.7
CBP films:									
**Ac-CNBPz**	0.1%	60	40	586	40.3	879	0.29	2.0	0.6
**Ac-CNBPzBr**		47	27	590	38.1	386	0.21	2.2	1.8
**Ac-CNBPz**	1%	55	37	596	41.6	529	0.24	2.0	1.1
**Ac-CNBPzBr**		50	31	602	39.2	194	0.20	2.2	3.6
**Ac-CNBPz**	8%	62	39	605	48.6	331	0.24	1.7	1.6
**Ac-CNBPzBr**		45	26	614	43.8	61	0.17	1.9	10.1
**Ac-CNBPz**	10%	71	50	619	62.7	272	0.23	1.3	1.7
**Ac-CNBPzBr**		50	26	628	58.1	18	0.18	1.4	30.1
**Ac-CNBPz**	15%	40	30	677	69.2	158	0.09	1.2	4.9
**Ac-CNBPzBr**		25	22	691	64.1	9	0.05	1.3	95.2
**Ac-CNBPz**	30%	19	12	701	79.6	11	0.14	0.4	11.8
**Ac-CNBPzBr**		11	9	715	75.7	3	0.02	1.2	322.3

[a] – Photoluminescence (PL) quantum yield, values recorded using integrating sphere: solutions in N_2-purged toluene, for CBP doped films values were corrected for vacuum conditions as follows: $\Phi_{PL} = \Phi_{PL\_air} \times (A_{vac}/A_{air})$, where Φ_{PL_air} is PL quantum yield obtained under oxygen atmosphere, A_{vac} and A_{air} are total areas under PL intensity decays measured in vacuum and under oxygen atmosphere, respectively.[b] – PL maxima, [c] – All of the presented photophysical parameters (τ_{PF}, τ_{DF}, k_r, k_{ISC}, k_{rISC}) were calculated from the PL intensity decay profiles (solutions: **Fig. S1**, CBP doped films: **Fig. 2C**, **Fig. S4C-D**). A detailed procedure for calculations is shown later in this section, equations S1-10.

Table S2. Steady-state emissive properties of **Ac-CNBPz** and **Ac-CNBPzBr** in toluene and 10% doped CBP films.

	Φ_{PL}	λ_{PL}	S_1 / λ_{onset}	T_1 / λ_{onset}	ΔE_{S1-T1}
	[%]	[nm]	[eV] / [nm]	[eV] / [nm]	[eV]
Solution (toluene)					
**Ac-CNBPz**	21	649	2.33 / 532	2.29 / 541	0.04 ± 0.01
**Ac-CNBPzBr**	11	658	2.30 / 539	2.28 / 543	0.02 ± 0.01
CBP films, 10%					
**Ac-CNBPz**	71	619	2.34 / 530	2.29 / 541	0.05 ± 0.01
**Ac-CNBPzBr**	50	628	2.31 / 537	2.28 / 542	0.03 ± 0.01

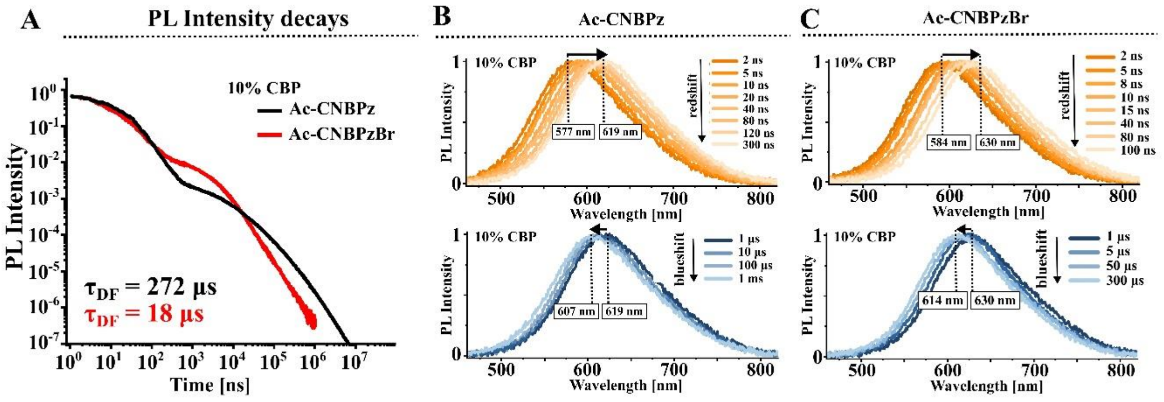

Figure S5. A: PL intensity decays of **Ac-CNBPz** and **Ac-CNBPzBr** in 10% CBP films at RT under vacuum, **B, C**: time-resolved spectra of **Ac-CNBPz** and **Ac-CNBPzBr**, respectively.

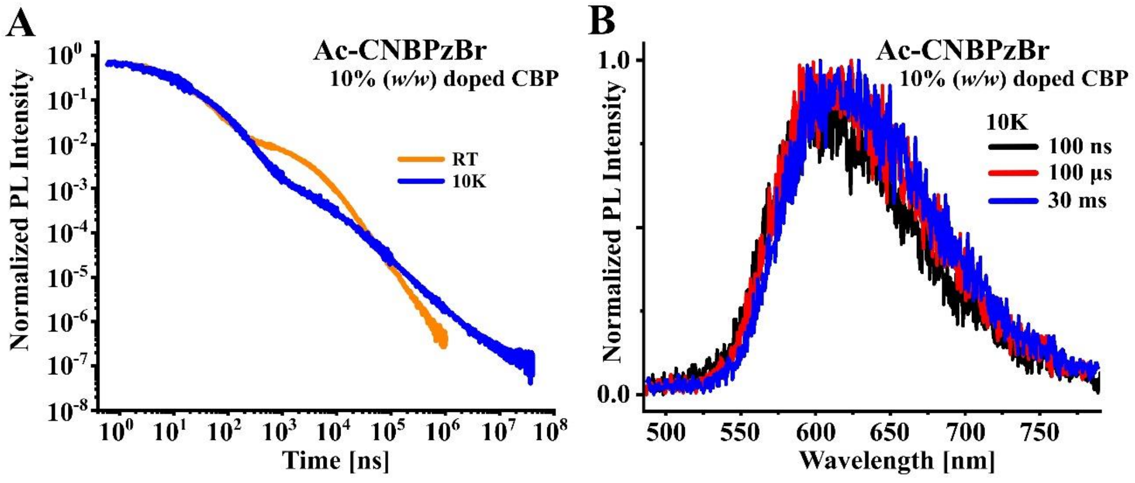

Figure S6. A: PL intensity decays of **Ac-CNBPzBr** at RT and 10K in CBP (10% *w/w*) doped films, **B**: Time-resolved emission spectra of **Ac-CNBPzBr** recorded with different time delays.

Determination of photophysical parameters

PL decay curves showed in **Figures 2C, 2F**, **4D** (main text) and **Figures S1, S4C, S4D, S5A,** were fitted with the multiexponential equation:

$$I(t) = A_0 + \sum_{i=1}^{n} A_i \exp\left(-{}^{t}/_{\tau_i}\right), \tag{S1}$$

where A_i is the pre-exponential factor, τ_i is the decay time and $I(t)$ is emission intensity. Average lifetimes of prompt (τ_{PF}) and delayed fluorescence (τ_{DF}) were determined using the following formula:

$$\tau_{PF,\ DF} = \sum_{i=1}^{n} f_i \tau_i, \tag{S2}$$

where $f_i$is fractional contribution of i-th component expressed as:

$$f_i = \frac{A_i \tau_i}{\sum_{i=1}^{n} A_i \tau_i}. \tag{S3}$$

The ratio of DF and PF quantum yields $\varphi_{DF}/\varphi_{PF}$ was determined as follows:

$$\frac{\varphi_{DF}}{\varphi_{PF}} = \frac{A_{DF}\tau_{DF}}{A_{PF}\tau_{PF}}, \tag{S4}$$

where A_{DF} and A_{PF} are pre-exponential factors of delayed and prompt fluorescence, respectively. Rate constants of radiative (k_r) and nonradiative (k_{nr}) decay and intersystem crossing (k_{ISC}) are given by equations[1]:

$$k_r = \frac{\varphi_{PF}}{\tau_{PF}}, \tag{S5}$$

$$k_{ISC} = \frac{\varphi_{DF}}{\varphi \tau_{PF}}, \tag{S6}$$

$$k_{nr} = \frac{1}{\tau_{PF}} - (k_r + k_{ISC}), \tag{S7}$$

where φ is PLQY ($\varphi_{DF} + \varphi_{PF}$). Further, the quantum yields for ISC and rISC are calculated as:

$$\varphi_{ISC} = k_{ISC}\tau_{PF}, \tag{S8}$$

$$\varphi_{rISC} = \frac{1 - \varphi_{PF}/\varphi}{\varphi_{ISC}}. \tag{S9}$$

Finally, rISC rate constant (k_{rISC}) can be calculated as:

$$k_{rISC} = \frac{\varphi_{rISC}}{\tau_{DF}}\left(\frac{\varphi}{\varphi_{PF}}\right). \tag{S10}$$

Photophysical parameters are presented in **Table S1**.

Section S2. OLEDs

Device fabrication.

The OLEDs were fabricated using patterned indium-tin-oxide-coated glass (VisionTek Systems) with a sheet resistance of 15 Ω sq^{-1}. After sonicating in acetone and isopropanol, oxygen-plasma-cleaned substrates were loaded into a Kurt J. Lesker Spectros II deposition chamber, and both small-molecule and cathode layers were thermally evaporated at a base pressure of 10^{-6} mbar. Each emitting layer was formed by multiple deposition of the compounds (emitter(s) and host) at a specific rates to obtain the particular content of the materials. The aluminum (Al) and lithium fluoride (LiF) depositions were conducted at 0.5 Å s^{-1} and 0.1 Å/s rate, respectively. The sizes of the pixels were 4, 8, and 16 mm^2.

Devices architecture.

DEV1, DEV2: ITO (100 nm)/HAT-CN (5 nm)/TAPC (70 nm)/CBP:TADF emitter (20 nm)/T2T (3 nm)/TPBi (50 nm)/LiF (0.8 nm)/Al (70 nm)

DEV3, DEV4, DEV5: ITO (100 nm)/HAT-CN (5 nm)/TAPC (70 nm)/CBP:TADF emitter:BPPC (30 nm)/T2T (3 nm)/TPBi (50 nm)/LiF (0.8 nm)/Al (70 nm)

Materials used for OLEDs fabrication.

For the device fabrication (**Fig. 3D**, main text), we used **HAT-CN** *(2,3,6,7,10,11-hexacyano-1,4,5,8,9,12-hexaazatriphenylene)* as hole injection layer and **TAPC** *(1,1-bis(4-di-p-tolylaminophenyl)cyclohexane)* as a hole transporting layer (HTL). The emissive layer (EML) consisted of host *4,4'-bis(N-carbazolyl)-1,10-biphenyl* (**CBP**) and respective emitter(s) in specific concentrations. **T2T** (*2,4,6-tris(biphenyl-3-yl)-1,3,5-triazine*) and **TPBi** *([2,2',2''-(1,3,5- benzinetriyl)-tris(1-phenyl-1-H-benzimidazole)])* were used as electron-transporting layers (ETL). Lithium fluoride (**LiF**) and aluminum (**Al**) were used as cathodes.

The materials for OLED fabrication were purchased from Sigma Aldrich or Lumtec and were purified by temperature-gradient sublimation in a vacuum.

Electroluminescence characterization.

The electroluminescent characteristics of the prepared devices were recorded using an integrating sphere (Labsphere) connected to a source measure unit (Keithley 2400) and an Ocean Optics QePro spectrometer.

Table S3. The electroluminescence performance of selected NIR OLEDs (715 – 840 nm) based on DA-type TADF molecules as emitters and hyperfluorescence systems, used in **Fig. 5D**.

#	compound	λ_{EL} [nm][a]	EQE_{max} [%]	Reference
		TADF NIR OLED		
1	**TCPQ**	718	5.4	[2]
2	**BF2**	721	9.7	[3]
3	**TPA-QCN**	733	3.9	[4]
4	**TPA-PZTCN**	734	13.4	[5]
5	**TPAM-BF2**	737	6.5	[6]
6	**TBSMCN**	750	14.3	[7]
7	**dpTPAAP**	752	6.8	[8]
8	**DBF2**	758	5.1	[9]
9	**DAD-1**	760	3.7	[10]
10	**TPAM-BF2**	762	2.3	[6]
11	**TPAAP**	765	5.1	[11]
12	**APDC-DTPA**	777	2.2	[12]
13	**DCN-PhTPA**	780	1.8	[13]
14	**SDPA-APDC**	782	2.6	[14]
15	**DCN-SPTPA**	800	2.6	[14]
16	**TBSMCN**	804	2.2	[7]
17	**DCPA-BBPA**	812	0.5	[15]
18	**OPDC-DTPA**	834	0.46	[16]
19	**DCPA-TPA**	838	0.58	[16]
		Hyperfluorescence NIR OLED		
20	**TPA-DCPP + TPA-ThQ**	780	3.8	[17]
21	**TPA-DCCP+BPPC**	790	5.4	[18]
22	**Ac-CNBPzBr + BPPC**	794	4.1	This work (DEV3)
23	**Ac-CNBPz + BPPC**	794	3.7	This work (DEV4)
24	**TPAM-BF2 + BPPC-Ph**	840	3.4	[6]

[a] – λ_{EL}: maximum of electroluminescence

Prediction of rISC rates using Marcus-Hush equation

Marcus-Hush equation[19]:

$$k_{\mathrm{rISC}} = \frac{V^2}{\hbar}\sqrt{\frac{\pi}{\mathrm{k_B T}\lambda}}\exp\left[-\frac{(\Delta E_{\mathrm{ST}} + \lambda)^2}{4\mathrm{k_B T}\lambda}\right], \tag{S11}$$

where

- V - SOC constant,
- $\hbar$ - reduced Planck's constant,
- λ - sum of internal and external reorganization energies for respective transition,
- ΔE_{ST} - the energy gap between S_1 and T_1.

Table S4. Electronic parameters used to calculate rate constant for rISC with Marcus-Hush equation.

	$E(S_1)^a$	$E(T_1)^a$	ΔE_{ST}	V^b	$\frac{V^2}{\hbar}\sqrt{\frac{\pi}{\mathrm{k_B T}\lambda}}$ [c]	$\exp\left[-\frac{(\Delta E_{\mathrm{ST}}+\lambda)^2}{4\mathrm{k_B T}\lambda}\right]$	k_{rISC}
	[eV]	[eV]	[eV]	[cm^{-1}]	[10^7 s^{-1}]		[10^7 s^{-1}]
**Ac-CNBPz**	2.34	2.29	0.05	0.44	12.86	0.11	1.65
**Ac-CNBPzBr**	2.31	2.28	0.03	0.47	16.92	0.19	3.26

[a] – Energies of respective excited states were obtained from steady-state PL spectra. [b] – SOC value calculated using ORCA software (details in section quantum chemical calculations). [c] – the reorganization energy value λ was assumed to be 0.1 eV.

Section S3. X-ray diffraction

Single-Crystal X-Ray Diffraction (SCXRD) measurements and structure refinement

SCXRD data were collected on an Oxford Diffraction Gemini R ULTRA Ruby CCD diffractometer with CuKα (λ = 1.5418 Å) radiation at T = 291(2) K (**Table S5**). The lattice parameters were obtained by least-squares fit to the optimised setting angles of the reflections collected by means of CrysAlis CCD. Data were reduced using CrysAlis RED software and applying multi-scan absorption corrections[20]. The structure of **Ac-CNBPzBr** was solved with direct methods that carried out refinements by full-matrix least-squares on F^2 using the SHELXL-2017/1 program[21]. All H-atoms bound to C-atoms were placed geometrically and refined using a riding model with $d_{(C–H)}$ = 0.93–0.97 Å and $U_{iso}(H) = 1.2U_{eq}(C)$ ($d_{(C–H)}$ = 0.96 Å and $U_{iso}(H) = 1.5U_{eq}(C)$ for the -CH_3 groups). The following programs were used to prepare the molecular graphics: ORTEPII, and Mercury[22 23].

Single-Crystal X-Ray Diffraction measurement shows that compound **Ac-CNBPzBr** crystallizes in triclinic *P*-1 space group as 1,4-dioxane solvate and is isostructural with 3-[4-(9,9-dimethylacridin-10(9H)-yl)phenyl]dibenzo[a,c]phenazine-11,12-dicarbonitrile (**Ac-CNBPz**)[24]. (**Figure S7A, S7B**)

In the crystal of the **Ac-CNBPzBr**, the acridine skeleton adopts a butterfly conformation, with the angle between the mean planes of the right- and left-hand halves of the acridine skeleton equals *ca.* 20°. In turn, the dihedral angle between acridine moiety and phenylene linker ($\theta_{D–\pi}$) is nearly 90°, whereas dihedral angle between phenylene linker and **CNBPz** ($\theta_{A–\pi}$) is about 40°. For comparison, in the crystal of **Ac-CNBPz** these angles are *ca.* 6, 78 and 43°, respectively.[27]

In the crystal packing, adjacent molecules of compound **Ac-CNBPzBr** are linked *via* short $N_{(nitrile)}\cdots Br$ contact (d(N···Br)=3.38 Å), $C_{(phenyl)}–H\cdots\pi_{(acridine)}$ interaction (d(H···π)=2.81 Å) and weak $C_{(acridine)}–H\cdots N_{(nitrile)}$ hydrogen bond (d(H···N)=2.76 Å) to form blocks along *b*-axis. The neighbouring blocks interact by π–π interaction between the planar, aromatic fragment of the molecule, including the phenazine skeleton, and as a result π–stacking dimers are observed.

In the dimer 1 (designated as D1), two aromatic rings of phenazine moiety (including the heteroaromatic phenazine ring) participate in π-stacking interactions and are arranged in face-to-face alignment (with separation of 3.50 Å between the mean planes of the phenazine moieties and distance between the centroids *ca.* 4 Å). Dimer D1 is also present in the crystal of **Ac-CNBPz**.

Table S5. Crystal data and structure refinement for **Ac-CNBPzBr**.

**Compound**	**1**
Chemical formula	$C_{47}H_{34}N_5O_2Br$
Formula weight/g·mol^{-1}	780.70
Crystal system	triclinic
Space group	*P*-1
a/Å	9.452(2)
b/Å	14.108(2)
c/Å	14.6102(12)
α/°	98.515(9)
β/°	96.615(13)
γ/°	100.263(16)
V/Å^3	1875.7(6)
Z	2
T/K	291(2)
Λ_{Cu}/Å	1.54184
ρ_{calc}/g·cm^{-3}	1.382
F(000)	804
μ/mm^{-1}	0.202
θ range/°	3.23 - 62.50
Completeness θ/%	99.8
Reflections collected	17425
Reflections unique	5957 [R_{int}=0.1950]
Data/restraints/parameters	5957 / 0 / 498
Goodness of fit on F^2	1.021
Final R_1 value (I>2σ(I))	0.0964
Final wR_2 value (I>2σ(I))	0.1990
Final R_1 value (all data)	0.2485
Final wR_2 value (all data)	0.2754
Largest diff. peak/hole/e Å^{-3}	0.464/-0.275
CCDC number	2385115

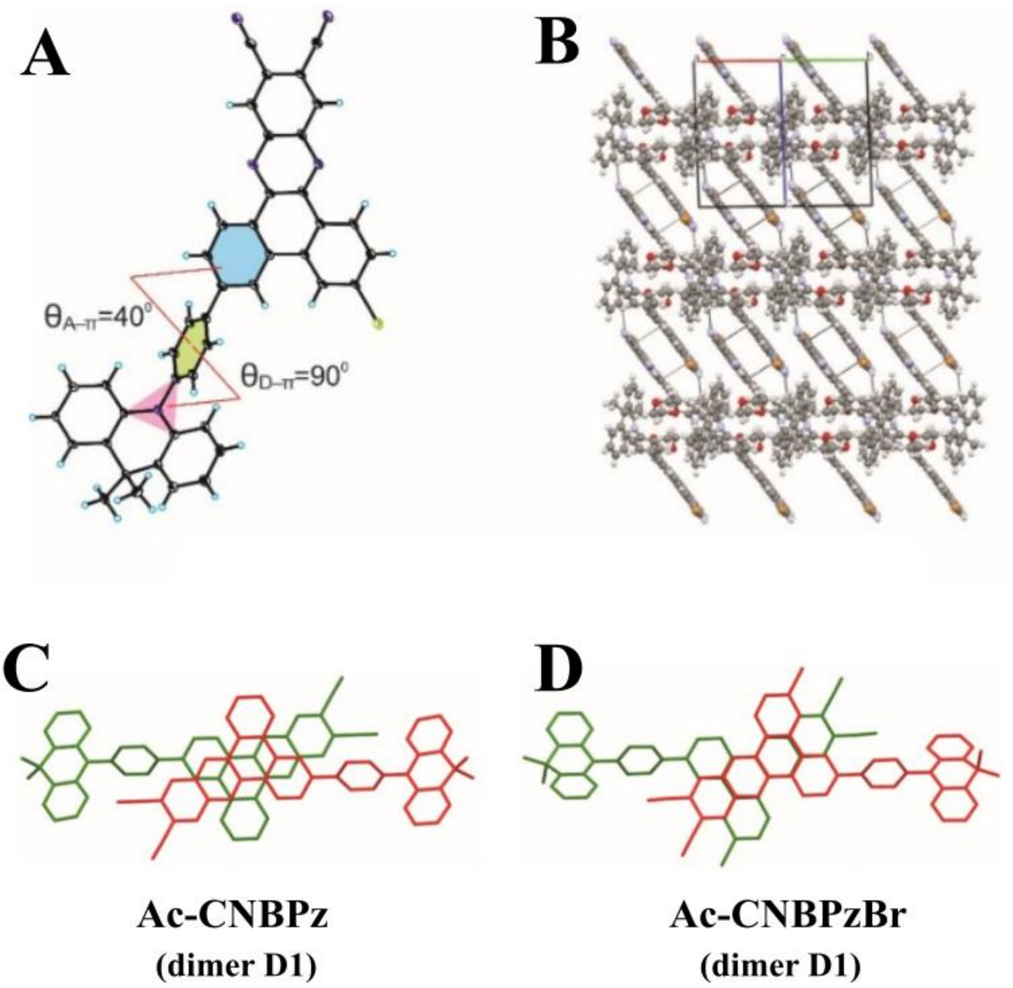

Figure S7. Molecular structure (**A**) and crystal packing (**B**) of **Ac-CNBPzBr**. Displacement ellipsoids are drawn at the 25% probability level. H atoms are shown as small spheres of arbitrary radius. The mean planes of donor, phenylene and acceptor are highlighted in pink, green, and blue, respectively. The arrangement of molecules in the dimer D1 crystals of the **Ac-CNBPz** (**C**) and **Ac-CNBPzBr** (**D**).

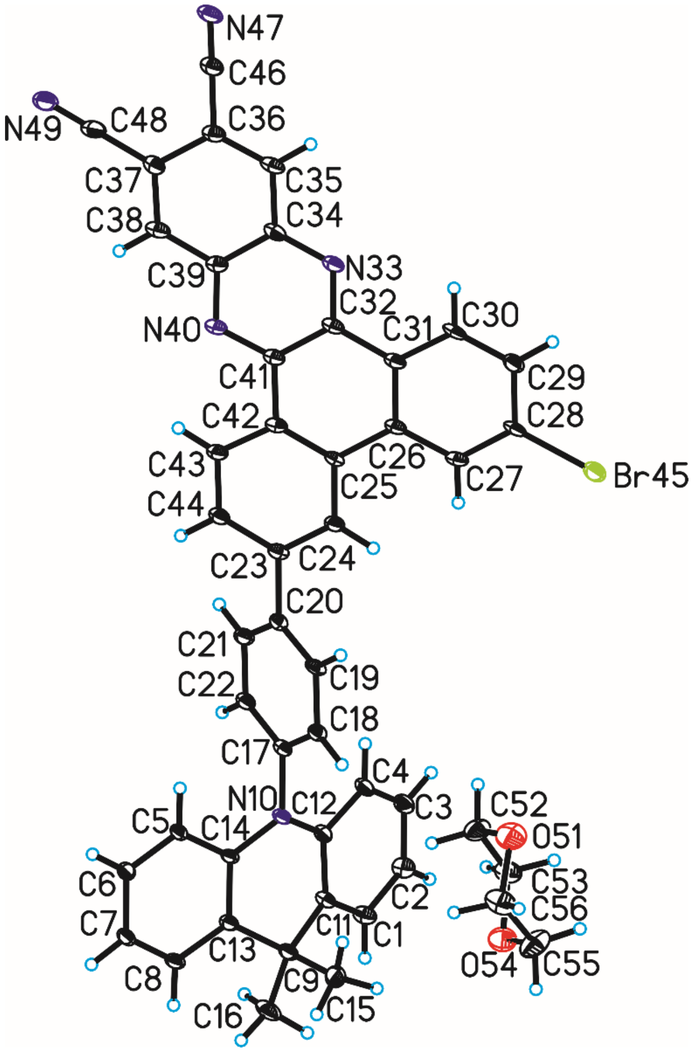

Figure S8. Molecular structure of the **Ac-CNBPzBr**, showing the atom-labelling scheme, (displacement ellipsoids are drawn at the 20% probability level, H atoms are shown as small spheres of arbitrary radius).

Section S4. Quantum chemical calculations

D1-type dimers found in the crystal phase

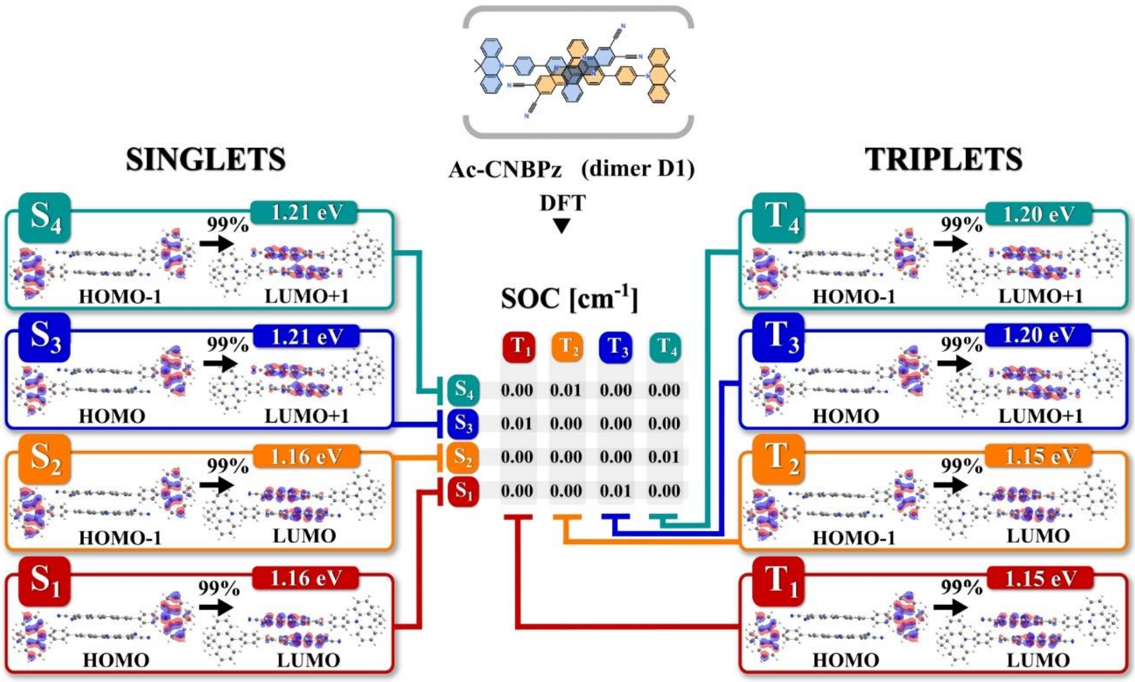

Figure S9. The energy levels diagram for dimer D1 of **Ac-CNBPz** with respective molecular orbitals forming intermolecular charge-transfer excited states calculated using DFT B3LYP/LANL2DZ method. The spin-orbit coupling (SOC) constant values for respective singlet-triplet transitions are presented in matrix-form, thus for example the transitions S_1-T_3, S_3-T_1, S_2-T_4, and S_4-T_2 have SOC = 0.01 cm^{-1}.

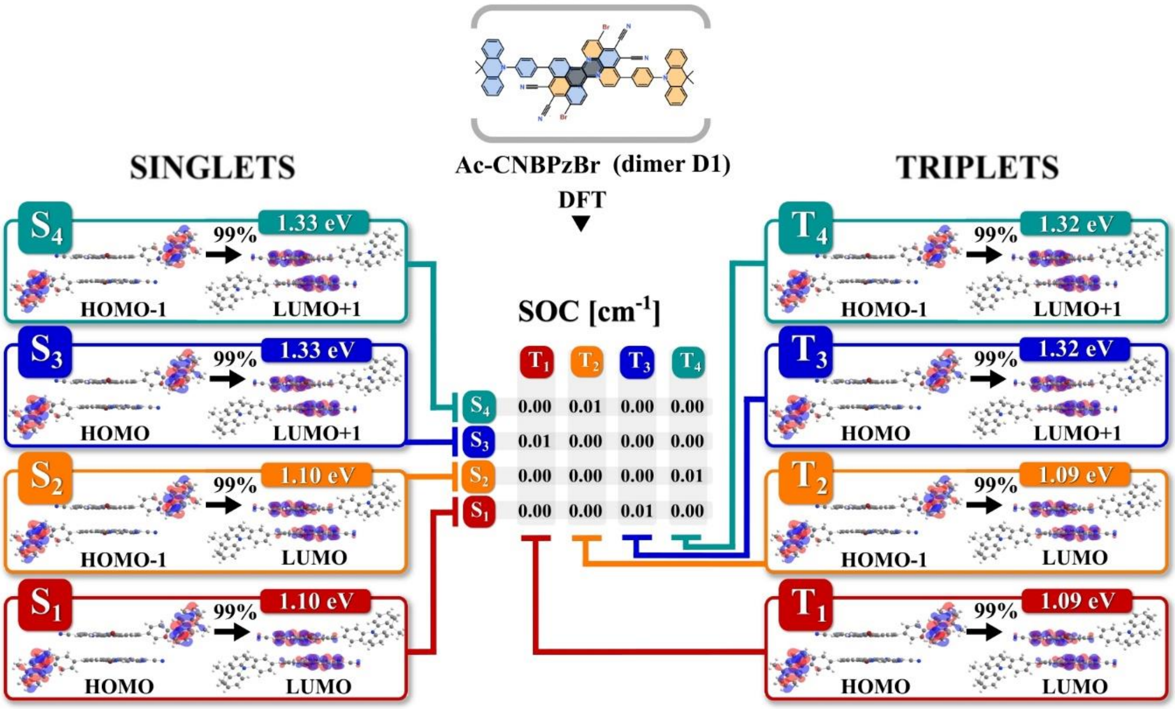

Figure S10. The energy levels diagram for dimer D1 of **Ac-CNBPzBr** with respective molecular orbitals forming intermolecular charge-transfer excited states calculated using DFT B3LYP/LANL2DZ method. The spin-orbit coupling (SOC) constant values for respective singlet-triplet transitions are presented in matrix-form, thus for example the transitions S_1-T_3, S_3-T_1, S_2-T_4, and S_4-T_2 have SOC = 0.01 cm^{-1}.

The electronic parameters of such dimers (D1) were analyzed on the B3LYP/LANL2DZ level of theory with a specific attention to the singlet-triplet transitions (**Fig. S9**, and **Fig. S10**, SI). Due to high symmetry and identical configuration of donor fragment, states S_1 and S_2, as well as T_1 and T_2 are degenerate, the same is true for higher energy states S_3 and S_4, as well as T_3 and T_4. Spin-orbit coupling between S_{1-4} and T_{1-4} pairs however does not exceed 0.01 cm^{-1}. This together with low oscillator strength of the singlet states (f <10^{-5}) explains the lack of TADF and emission in general in such dimers of both compounds in crystals. However, such dimers of **Ac-CNBPz** and **Ac-CNBPzBr**, presented in **Fig. 6A, Fig. S9**, and **Fig. S10**, cannot be responsible for TADF in films.

Other possible types of dimers

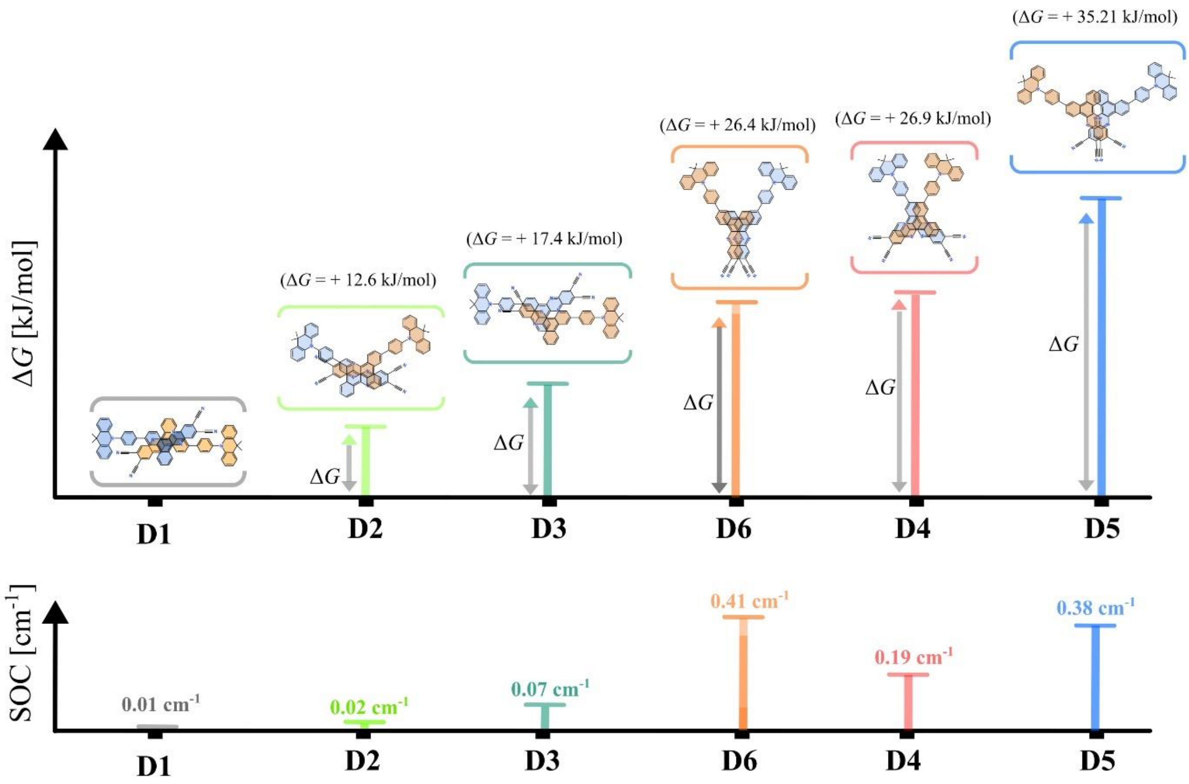

Figure S11. Dimers of **Ac-CNBPz** in their S_0-optimized geometry using M062X/LANL2DZ method with the Gibbs free energy of formation (Δ*G*) and spin-orbit coupling (SOC) constants for the low-energy singlet – triplet transitions.

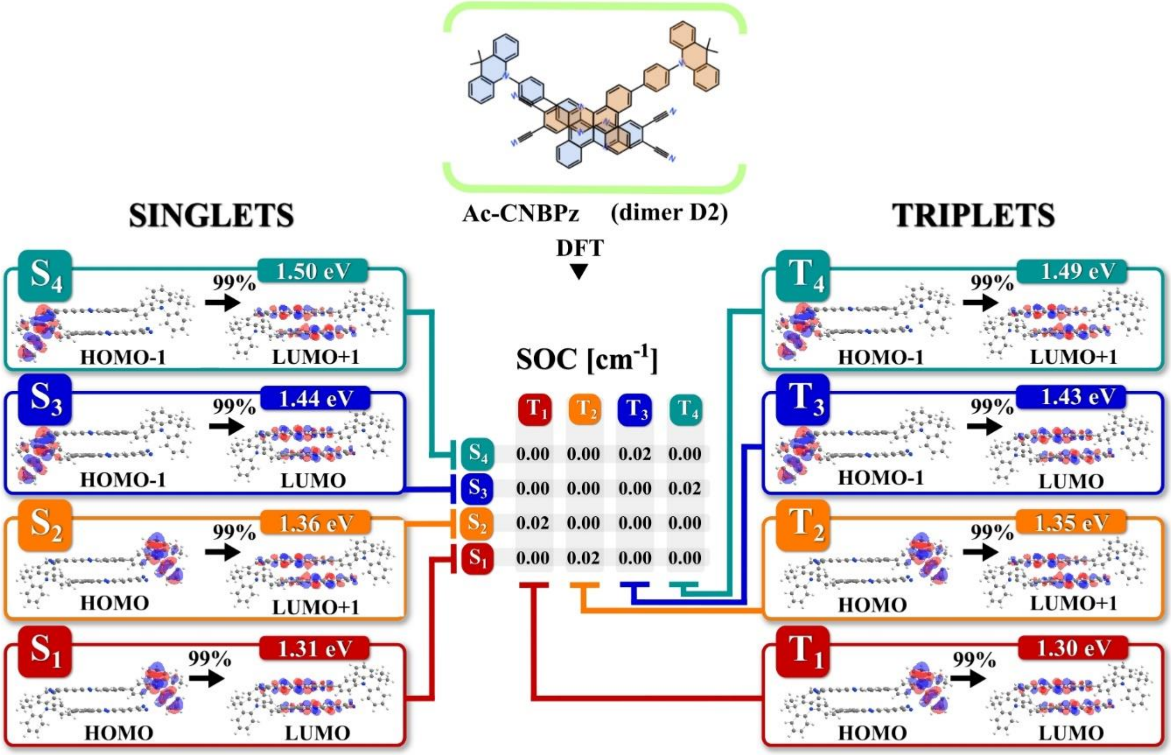

Figure S12. The energy levels diagram for dimer D2 of **Ac-CNBPz** with respective molecular orbitals forming intermolecular charge-transfer excited states calculated using DFT B3LYP/LANL2DZ method. The spin-orbit coupling (SOC) constant values are shown in the table at the center.

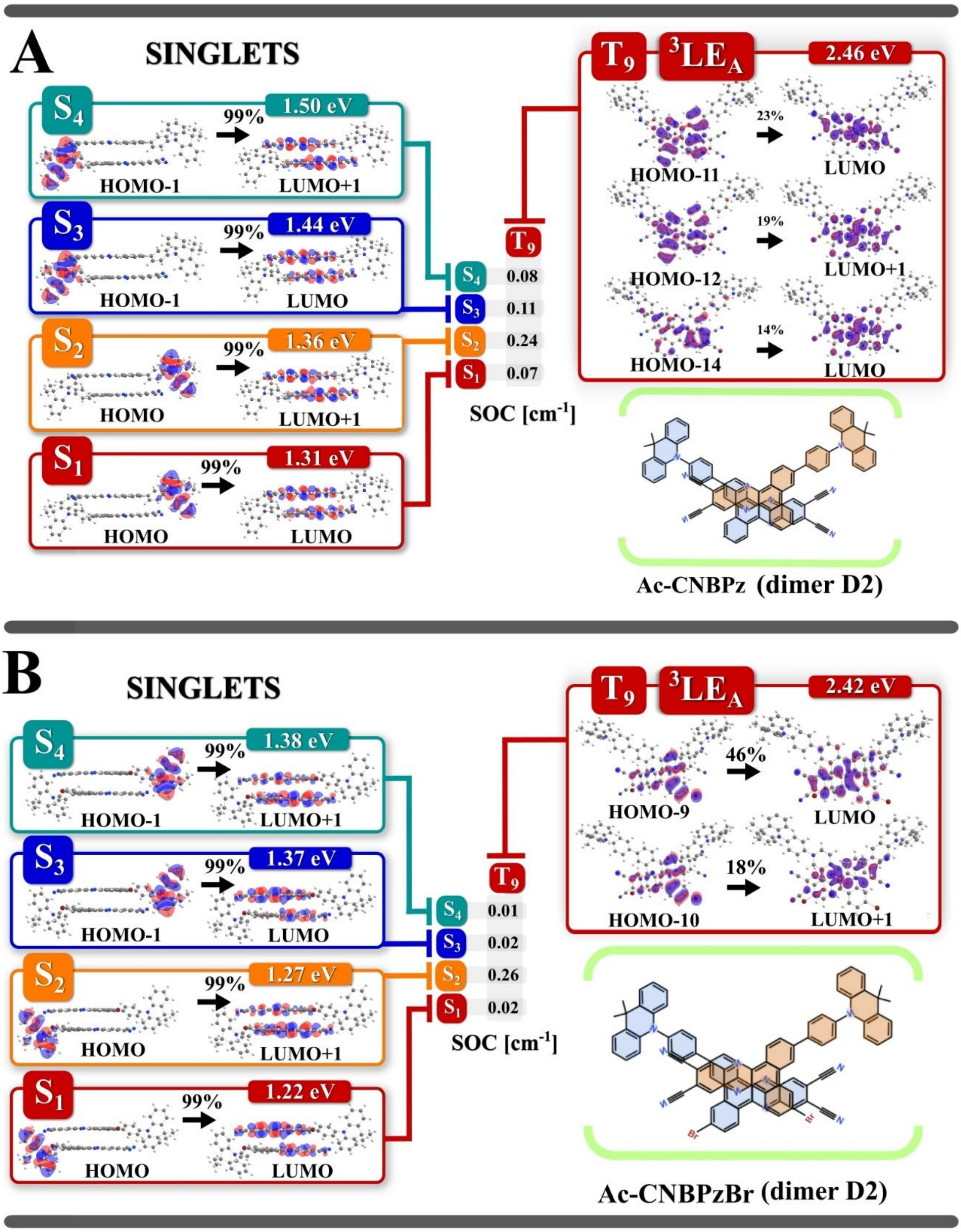

Figure S13. The energy levels diagram for dimer D2 of **Ac-CNBPz** (**A**) and **Ac-CNBPzBr** (**B**) with respective molecular orbitals forming intermolecular charge-transfer and locally-excited states calculated using DFT B3LYP/LANL2DZ method. The spin-orbit coupling (SOC) constant values are shown in the table at the center.

Section S5. NMR, MALDI-TOF spectra of emitters and synthetic procedures

Synthesis. The synthesis of the target compound **Ac-CNBPzBr** was accomplished in two steps. The first step involved a Suzuki coupling reaction catalyzed by palladium. In this key transformation, 3,6-dibromophenanthrene was reacted with the known compound 9,9-dimethyl-10-(4-(4,4,5,5-tetramethyl-1,3,2-dioxaborolan-2-yl)phenyl)-9,10-dihydroacridine.[27] reaction proceeded efficiently yielding the intermediate product **3**, which retains a bromine atom in its structure. Finally, **Ac-CNBPzBr** was obtained by cyclization reaction of **3** with 4,5-diaminophthalonitrile.

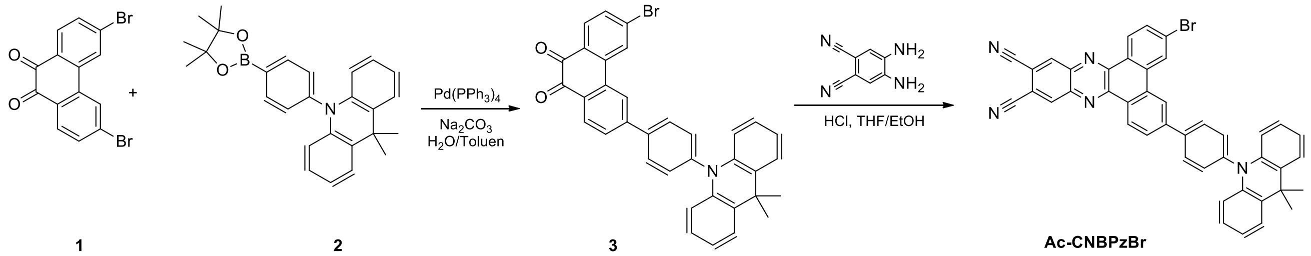

Scheme S1. Synthetic route for the preparation of **Ac-CNBPzBr**.

All solvents and reagents were purchased commercially and employed without further purification, in line with standard protocols. The compound 9,9-dimethyl-10-(4-(4,4,5,5-tetramethyl-1,3,2-dioxaborolan-2-yl)phenyl)-9,10-dihydroacridine (**2**) was synthesized according to procedures previously reported in the literature, confirming its structure and properties as described.[27]

The ^1H NMR spectra were recorded on a Bruker AVANCE III 500 instrument (500.13/125.76 MHz) with $CDCl_3$ as the solvent. Chemical shifts were recorded in parts per million(ppm) relative to tetramethylsilane (Me_4Si) as an internal reference. To determine the molecular mass, positive-ion mode MALDI-TOF analyses were performed on a Bruker Biflex III mass spectrometer, with 2,5-dihydroxybenzoic acid used as the matrix. The purification process was performed by column chromatography using silica gel GE60, 60-200μm (70-230 mesh), 60 Å, ensuring the isolation of the target compound.

Synthesis of *3-bromo-6-(4-(9,9-dimethylacridin-10(9H)-yl)phenyl)phenanthrene-9,10-dione* (**3**)

To a solution containing 3,6-dibromophenanthrene-9,10-dione (**1**) (0.37 g, 1 mmol) and 9,9-dimethyl-10-(4-(4,4,5,5-tetramethyl-1,3,2-dioxaborolan-2-yl)phenyl)-9,10-dihydroacridine (**2**) (0.21 g, 0.5 mmol) in dry toluene (15 mL), an aqueous solution of Na_2CO_3 (2.0 M, 7 mL) was added. Subsequently, $Pd(PPh_3)_4$ (12 mg, 0.01 mmol) was introduced to the reaction mixture. The reaction was carried out in a Schlenk flask under argon atmosphere at 70 ^oC for 24 hours. After the completion of the reaction, the solvent was evaporated under reduced pressure. The residue was then treated with 0.1 M HCl in water and extracted with dichloromethane. The resulting crude product was purified by column chromatography (eluent: petroleum ether/chloroform = 3:1, v/v) to isolate the desired compound in pure form, yielding **3** as an orange solid (0.48 g, 67% yield).
^1H NMR (500 MHz, $CDCl_3$) δ ppm 1.72 (s, 6 H), 6.35 (dd, *J*=7.81, *J*=0.98, 2 H), 6.97 (td, *J*=7.32, *J*=0.98, 2 H), 7.03 (dd, *J*=8.30, *J*=1.46, 2 H), 7.49 (dd, *J*=7.32, 1.46 Hz, 2 H), 7.53 (d, *J*=8.30 Hz, 2 H), 7.67 (dd, *J*=8.30, 1.46 Hz, 1 H), 7.84 (dd, *J*=8.30, 0.98 Hz, 1 H), 7.97 (d, *J*=8.30 Hz, 2 H), 8.11 (d, *J*=8.30 Hz, 1 H), 8.25 (d, 1 H), 8.32 (d, *J*=1.46 Hz, 1 H), 8.35 (d, *J*=8.30 Hz, 1 H); MALDITOF-MS: *m/z* calcd for $C_{35}H_{24}NO_2Br$ 571.1, found 556.1 $[M – CH_3]^+$.

Synthesis of *3-bromo-6-(4-(9,9-dimethylacridin-10(9H)-yl)phenyl)dibenzo[a,c]phenazine-11,12-dicarbonitrile* (**Ac-CNBPzBr**)

Compound **3** (40 mg, 0.056 mmol), 4,5-diaminophthalonitrile (10 mg, 0.063 mmol) and $HCl_{conc.}$ (10 μL) were dissolved in a mixture of tetrahydrofuran with ethanol (1:1, *v*/*v*, 35 mL) and stirred at room temperature. The reaction was monitored by TLC, and upon completion, the solvent was removed under reduced pressure. The resulting residue was purified *via* silica gel column chromatography (eluent: petroleum ether/chloroform = 2:1, v/v) followed by recrystallization from chloroform, yielding **Ac-CNBPzBr** as a dark orange powder (34 mg, yield 90%). ^1H NMR (500 MHz, $CDCl_3$) δ ppm 1.73 (s, 6 H), 6.41 (d, *J*=7.81 Hz, 2 H), 6.97 (td, *J*=7.32, *J*=0.98 Hz, 2 H), 7.03 (td, *J*=8.30, *J*=1.46 Hz, 2 H), 7.50 (dd, *J*=7.81, 1.46 Hz, 2 H), 7.57 (m, *J*=8.30 Hz, 2 H), 7.95 (dd, *J*=8.79, 1.46 Hz, 1 H), 8.09 (m, *J*=8.30 Hz, 2 H), 8.16 (dd, *J*=8.30 Hz, *J*=1.46 Hz, 1 H), 8.78 (s, 1 H), 8.83 (m, 3 H), 9.25 (d, *J*=8.30 Hz, 1 H), 9.47 (d, *J*=8.30 Hz, 1 H); MALDITOF-MS: *m/z* calcd for $C_{43}H_{26}N_5Br$ 691.14, found 676.13 $[M – CH_3]^+$.

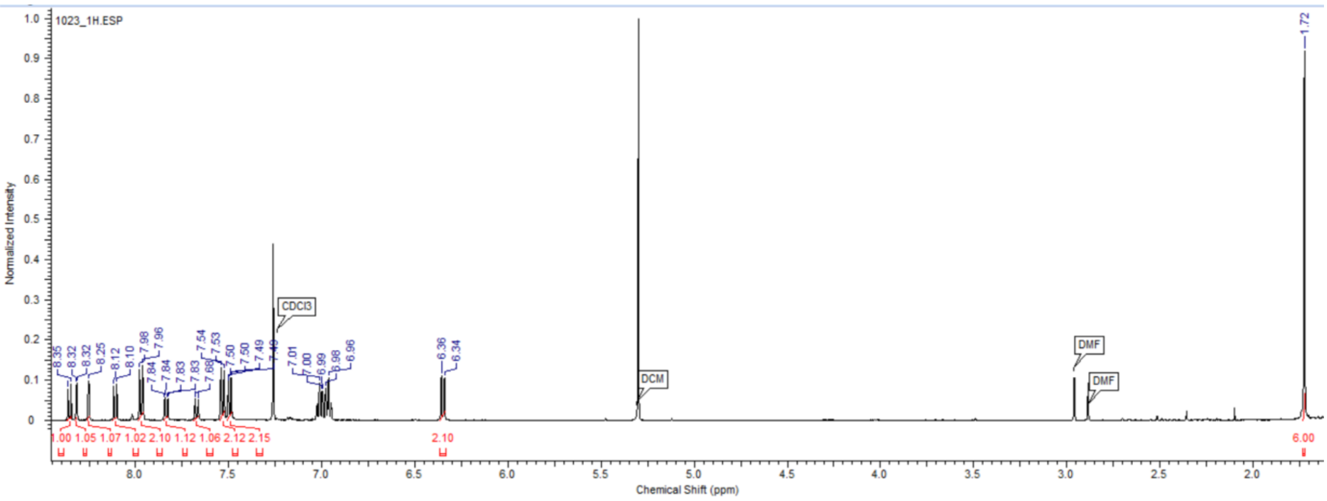

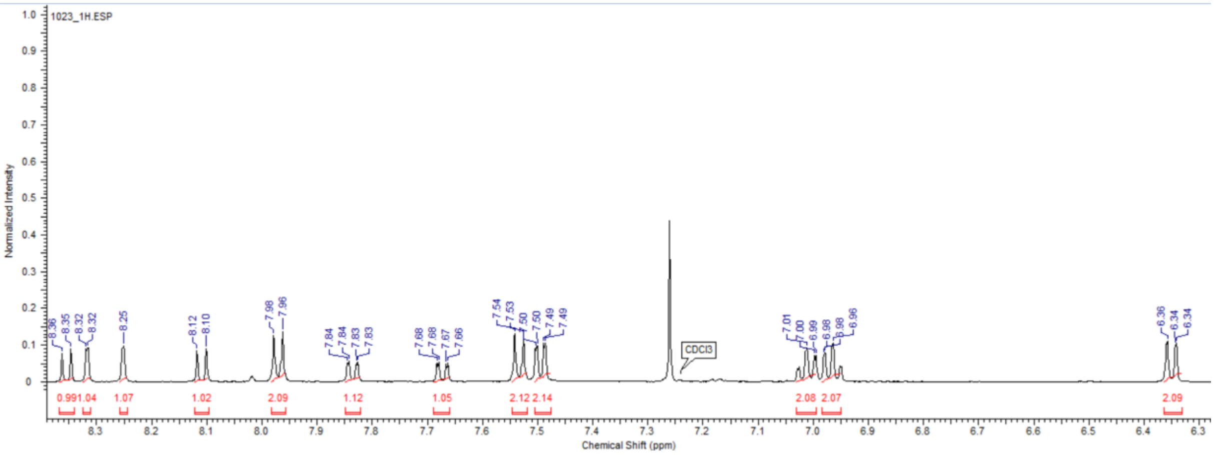

Figure S14. ^{1}H NMR spectrum of 3-bromo-6-(4-(9,9-dimethylacridin-10(9*H*)-yl)phenyl)-phenanthrene-9,10-dione (**3**) in $CDCl_3$.

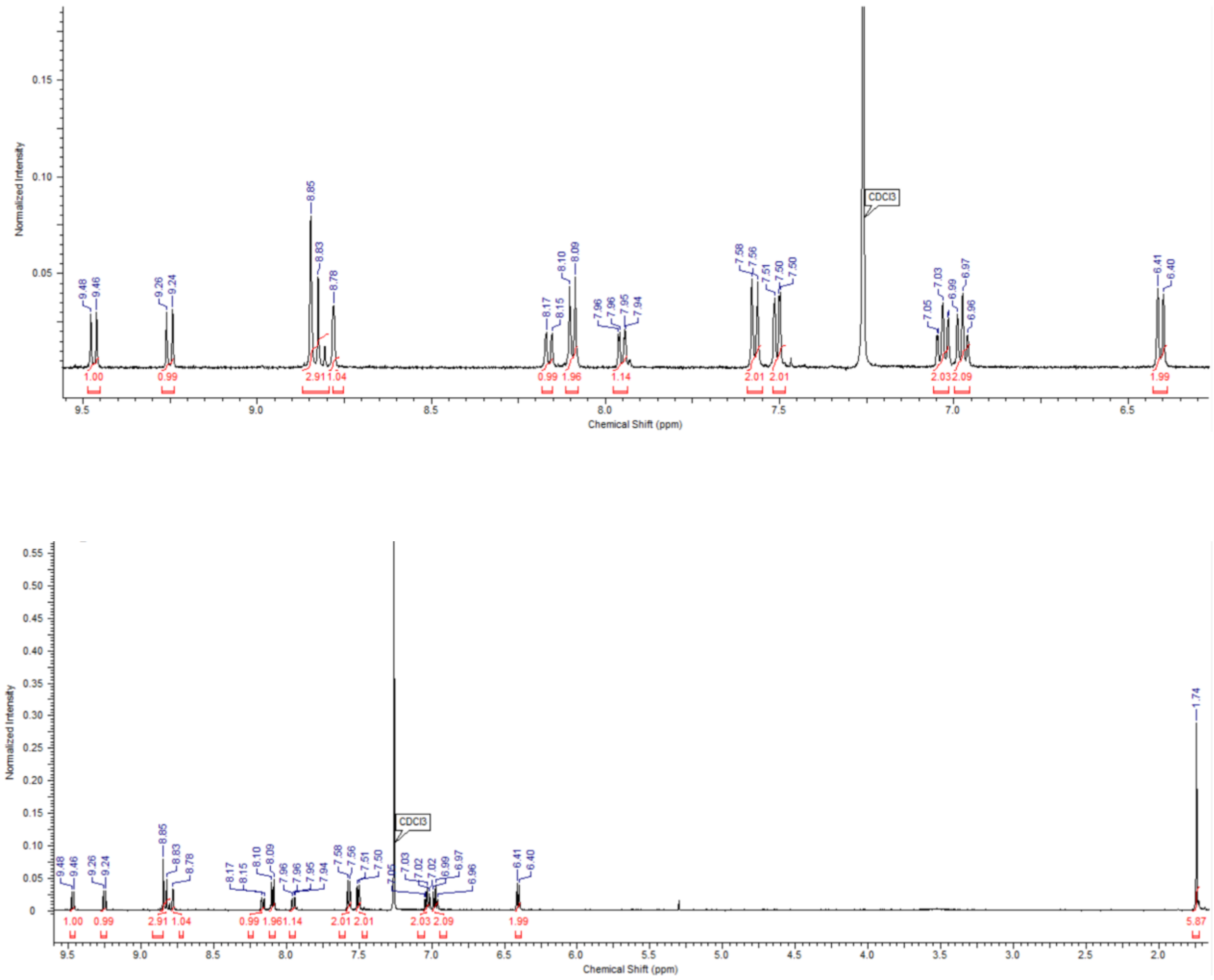

Figure S15. ^{1}H NMR spectrum of 3-bromo-6-(4-(9,9-dimethylacridin-10(9*H*)-yl)phenyl)dibenzo[*a*,*c*]phenazine-11,12-dicarbonitrile (**Ac-CNBPzBr**).

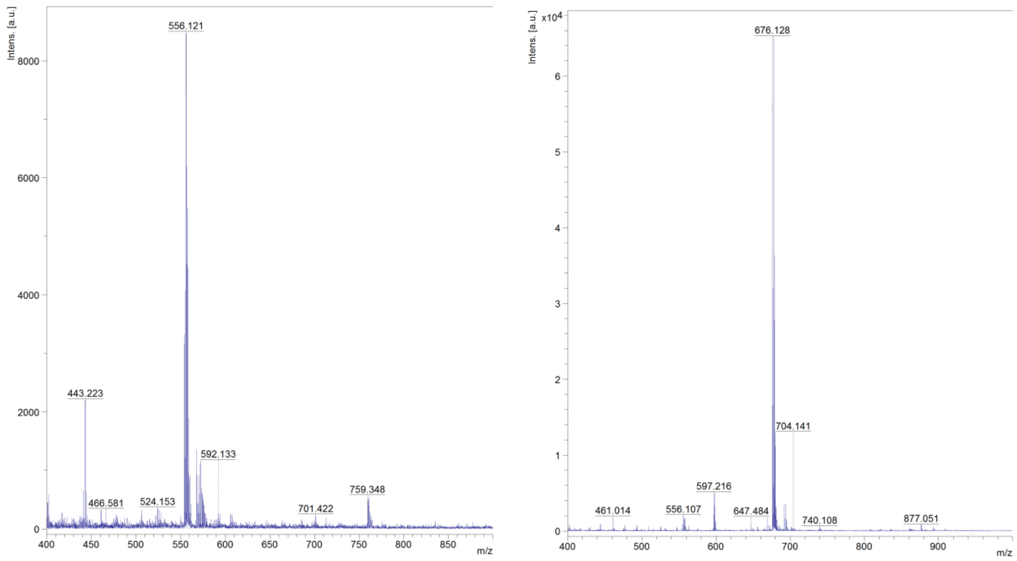

Figure S16. MALDI-TOF mass spectra of **3** and **Ac-CNBPzBr**, respectively.

REFERENCES

[1] Tao, Y., Yuan, K., Chen, T., Xu, P., Li, H., Chen, R., Zheng, C., Zhang, L., Huang, W. Thermally Activated Delayed Fluorescence Materials Towards The Breakthrough Of Organoelectronics. *Adv. Mater*. **26**, 7931 – 7958 (2015).

[2] Liu, Y., Yang, J., Mao, Z., Chen, X., Yang, Z., Ge, X., Peng, X., Zhao, J., Su, S-J., Chi, Z. Asymmetric Thermally Activated Delayed Fluorescence Emitter for Highly Efficient Red/Near-Infrared Organic Light-Emitting Diodes. *ACS Appl. Mater. Interfaces* **14**, 33606 – 33613 (2022).

[3] Kim, D.-H., A. D'Aléo, Chen, X.-K., Sandanayaka, A. D. S., Yao, D., Zhao, L., Komino, T., Zaborova, E., Canard, G., Tsuchiya, T., Choi, E., Wu, J. W., Fages, F., Bredas, J.-L., Ribierre, J.-C., Adachi, C. High-efficiency electroluminescence and amplified spontaneous emission from a thermally activated delayed fluorescent near-infrared emitter. *Nat. Photonics* **12**, 98–104 (2018).

[4] Li, C., Duan, R., Liang, B., Han, G., Wang, S., Ye, K., Liu, Y., Yi, Y., Wang, Y. Deep-Red to Near-Infrared Thermally Activated Delayed Fluorescence in Organic Solid Films and Electroluminescent Devices. *Angew. Chem., Int. Ed.* **56**, 11525–11529 (2017).

[5] Balijapalli, U., Nagata, R., Yamada, N., Nakanotani, H., Tanaka, M., D'Aléo, S., Placide, V., Mamada, M., Tsuchiya, Y., Adachi, C. Highly Efficient Near-Infrared Electrofluorescence from a Thermally Activated Delayed Fluorescence Molecule. *Angew. Chem. Int. Ed.* **60**, 8477-8482 (2021).

[6] Shahalizad A., Malinge, A., Hu, L., Haeberlé,L., Myers, D. M., Mao, J., Skene, W. G., Kéna-Cohen, S. Efficient Solution-Processed Hyperfluorescent OLEDs with Spectrally Narrow Emission at 840 nm. *Adv. Funct. Mater.* **31**, 2007119 (2021).

[7] Yu, Y., Xing, H., Liu, D., Zhao, M., Sung, H. H.-Y., Williams, I. D., Lam, J. W. Y., Xie, G., Zhao, Z., Tang, B. Z. Solution-processed AIEgen NIR OLEDs with EQE Approaching 15 %. . *Angew. Chem. Int. Ed.* **61**, 202204279 (2022).

[8] Xue, J., Xu, J., Ren, J., Liang, Q., Ou, Q., Wang, R., Shuai, Z., Qiao, J. Intermolecular charge-transfer aggregates enable high-efficiency near-infrared emissions by nonadiabatic coupling suppression. *Science China Chemistry* **65**, 1786-1795 (2021).

[9] Ye, H., Kim, D.-H., Chen, X.-K., Sandanayaka, A. D. S., Kim, J. U., Zaborova, E.,Canard, G., Tsuchiya, T., Choi, E., Wu, J. W., Fages, F., Bredas, J.-L., A. D'Aléo, Ribierre, J.-C., Adachi. Near-Infrared Electroluminescence and Low Threshold Amplified Spontaneous Emission above 800 nm from a Thermally Activated Delayed Fluorescent Emitter. *Chem. Mater.* **30**, 6702–6710 (2018).

[10] Kage, Y., Kang, S., Mori, S., Mamada, M., Adachi, C., Kim, D., Furuta, H. An Electron-Accepting aza-BODIPY-Based Donor–Acceptor–Donor Architecture for Bright NIR Emission. *Chem. Eur. J.* **27**, 5259-5267 (2021).

[11] Xue, J., Liang, Q., Wang, R., Hou, J., Li, W., Peng, Q., Shuai, Z., Qiao, J. Highly Efficient Thermally Activated Delayed Fluorescence via J-Aggregates with Strong Intermolecular Charge Transfer, *Adv. Mater.* **31**, 1808242 (2019).

[12] Yuan, Y., Hu, Y., Zhang, Y.-X., Lin, J.-D., Wang, Y.-K., Jiang, Z.-Q., Liao, L.-S., Lee, S.-T. Over 10% EQE Near-Infrared Electroluminescence Based on a Thermally Activated Delayed Fluorescence Emitter. *Adv. Funct. Mater.* **27**, 1700986 (2017).

[13] Wang, H., Wang, K., Chen, J.-X., Zhang, X., Zhou, L., Fan, X.-C., Cheng, Y.-C., Hao, X.-Y., Yu, J., Zhang, X.-H. Enabling Record-high Deep-Red/Near-Infrared Electroluminescence Through Subtly Managing Intermolecular Interactions of a Thermally Activated Delayed Fluorescence Emitter. *Adv. Funct. Mater*. **33**, 2304398 (2023).

[14] Liu, J.-F., Wang, X.-Q., Yu, Y.-J., Zou, S.-N., Yang, S.-Y., Jiang, Z.-Q., Liao, L.-S. Highly efficient near-infrared thermally activated delayed fluorescence material based on a spirobifluorene decorated donor. *Org. Electron.* **91**, 106088 (2021).

[15] Yu, Y.-J., Hu, Y., Yang, S.-Y., Luo, W., Yuan, Y., Peng, C.-C., Liu, J.-F., Khan, A., Jiang, Z.-Q., Liao, L.-S. Near-Infrared Electroluminescence beyond 800 nm with High Efficiency and Radiance from Anthracene Cored Emitters**.** *Angew. Chem., Int. Ed.* **59**, 21578–21584 (2020).

[16] Ma, B., Ding, Z., Liu, D., Zhou, Z., Zhang, K., Dang, D., Zhang, S., Su, S.-J., Zhu, W., Liu, Y. A Feasible Strategy for a Highly Efficient Thermally Activated Delayed Fluorescence Emitter Over 900 nm Based on Phenalenone Derivatives. *Chem. Eur. J.* **29**, 202301197 (2023).

[17] Yamanaka, T., Nakanotani, H., Hara, S., Hirohata, T., Adachi, C. Near-infrared organic light-emitting diodes for biosensing with high operating stability. *Appl. Phys. Express* **10**, 074101 (2017).

[18] Brodeur, J., Hu, L., Malinge, A., Eizner, E., Skene, W. G., Kena-Cohen, S. Highly Efficient and Spectrally Narrow Near-Infrared Fluorescent OLEDs Using a TADF-Sensitized Cyanine Dye. *Adv. Opt. Mater.* **7,** 1901144 (2019).

[19] Samanta, P. K., Kim, D., Coropceanu, V., Brédas, J.-L. Up-Conversion Intersystem Crossing Rates in Organic Emitters for Thermally Activated Delayed Fluorescence: Impact of the Nature of Singlet vs Triplet Excited States. *J. Am. Chem. Soc.* ***139***, 4042– 4051 (2017).

[20] CrysAlis CCD and CrysAlis RED. Version 1.171.36.24. Oxford Diffraction Ltd. (Yarnton, 2012).

[21] Sheldrick, G. M. Crystal structure refinement with SHELXL. *Acta Crystallogr., Sect. C: Struct. Chem*. **71**, 3–8 (2015).

[22] Johnson, C. K. ORTEP II, Report ORNL-5138, Oak Ridge National Laboratory, Oak Ridge, TN, USA, (1976).

[23]Macrae, C. F. et al. Mercury 4.0: From visualization to analysis, design and prediction. *J. Appl. Crystallogr*. **53**, 226 (2020).

[24] Furue, R., Matsuo, K., Ashikari, Y., Ooka, H., Amanokura, N., Yasuda, T. Highly Efficient Red–Orange Delayed Fluorescence Emitters Based on Strong π-Accepting Dibenzophenazine and Dibenzoquinoxaline Cores: toward a Rational Pure-Red OLED Design. *Adv. Opt. Mater*. **6**, 1701147 (2018).
